# Supplementary material for: Stillbirth, neonatal and maternal mortality among caesarean births in Kenya and Uganda: a register-based prospective cohort study
Source: BMJ Open. 2022 Apr 6;12(4):e055904. doi: 10.1136/bmjopen-2021-055904 (PMC8987792; doi:10.1136/bmjopen-2021-055904)
Supplement: Supplementary data [file bmjopen-2021-055904supp001.pdf]

Online supplement

The hypothesized relationship is between cesarean section and maternal mortality as shown by the thick red arrow in the online supplementary figure S1. To keep the conceptual framework simple, we are showing the Directed Acyclic Graph (DAG) for one outcome only. We considered each potential confounder and discussed all the backdoor pathways connecting the exposure (cesarean section) to the outcome (maternal mortality) through the confounders. In other words, once that confounder is adjusted, those pathways will no longer exist, potentially eliminating confounding, estimating only the *direct effect* between exposure and the outcome. We assume that there is no confounder misclassification i.e., after adjustment for the confounder in the model there is no residual confounding left from that confounder. Further, the majority of these confounders are categorical, and in accordance with their biological relationship with the exposure and the outcome. Hence, their functional form is assumed to be adequate to account for confounding.

1. Adjusting for *multiple gestation* would eliminate all the backdoor paths connecting mortality to cesarean section, through PTBi intervention. The backdoor paths from viability and birth weight/gestational age (GA) through multiple gestation are also removed. In the backdoor path MMR – multiple gestation – cesarean section – birth weight/GA, cesarean section is a collider. Adjustment for multiple gestation is necessary because multiple gestation pregnancy is a clinical indication for cesarean section. However, this could lead to over adjustment by inflating the standard error, as cesarean section is a collider in this pathway. We will still include multiple gestation in the preliminary model.
2. Adjusting for *birth weight/GA* would eliminate the direct path from birth weight/ GA to cesarean section and the backdoor paths through PTBi intervention, maternal history (e.g., age, prior cesarean section), viability, complications that leads to maternal deaths. The pathway from birth weight/GA – complications – maternal history leading on to maternal deaths through several other variables in the DAG are blocked paths because complication is a collider. Irrespective, birth weight adjustment removes the direct path from cesarean section – birth weight/GA – complications and will be included in the model.
3. Adjusting for *delivery volume*, will remove the backdoor from MMR – delivery volume – cesarean section. Further, it removes two additional directed paths from delivery volume to facility type and country and will be included in the model.
4. *PTBi intervention* requires adjustment because of the backdoor path from maternal death – PTBi – cesarean section. It will also remove the directed paths from PTBi intervention – facility readiness, PTBi intervention – APGAR score, facility type – PTBi intervention and country – PTBi intervention.
5. *Facility type* requires adjustment for the backdoor path maternal death – facility type – cesarean section. This adjustment will also remove the directed path from country – facility type.
6. The last confounder necessary to adjust is *country* for the backdoor path MMR – country – cesarean section.
7. *Maternal complications* will determine the need for a cesarean section. Independent of those cases that require a cesarean section, maternal complications such as PPH are primary reasons for maternal deaths. Complete maternal complication data is unavailable and hence it is a limitation. Statistically it leaves the potential for residual confounding.
8. A close look at this stage of the model will reveal that *APGAR score* does not require adjustment. All the backdoor paths from maternal death to cesarean section are through APGAR and stillbirth, making APGAR a collider and all those are blocked paths.
9. All other paths from viability, *weekend delivery and facility readiness* are blocked paths where cesarean section is the collider. Hence, conceptually there is no need for adjustment of any other confounder.
10. Of birth weight and GA, the final model had birth weight and not both because including GA leads to about loss of 8000 births, both are colinear (Pearson's correlation = 0.5) and the AICs for both models are close (AIC for the birth weight model is 1779 and AIC for the GA model is 1693).
11. Statistically adjusting for multiple gestation did not change the exposure – outcome association. The association with MMR presented in table 2 changed to 3.31 (2.21, 4.97) after including multiple gestation in the reported model. Hence, multiple gestation was not retained in the final model.

Ghosh R, *et al. BMJ Open* 2022; 12:e055904. doi: 10.1136/bmjopen-2021-055904

Figure S1. Conceptual diagram describing the hypothesized relationship (red arrow) between cesarean section and maternal mortality and the potential confounding due to other factors.

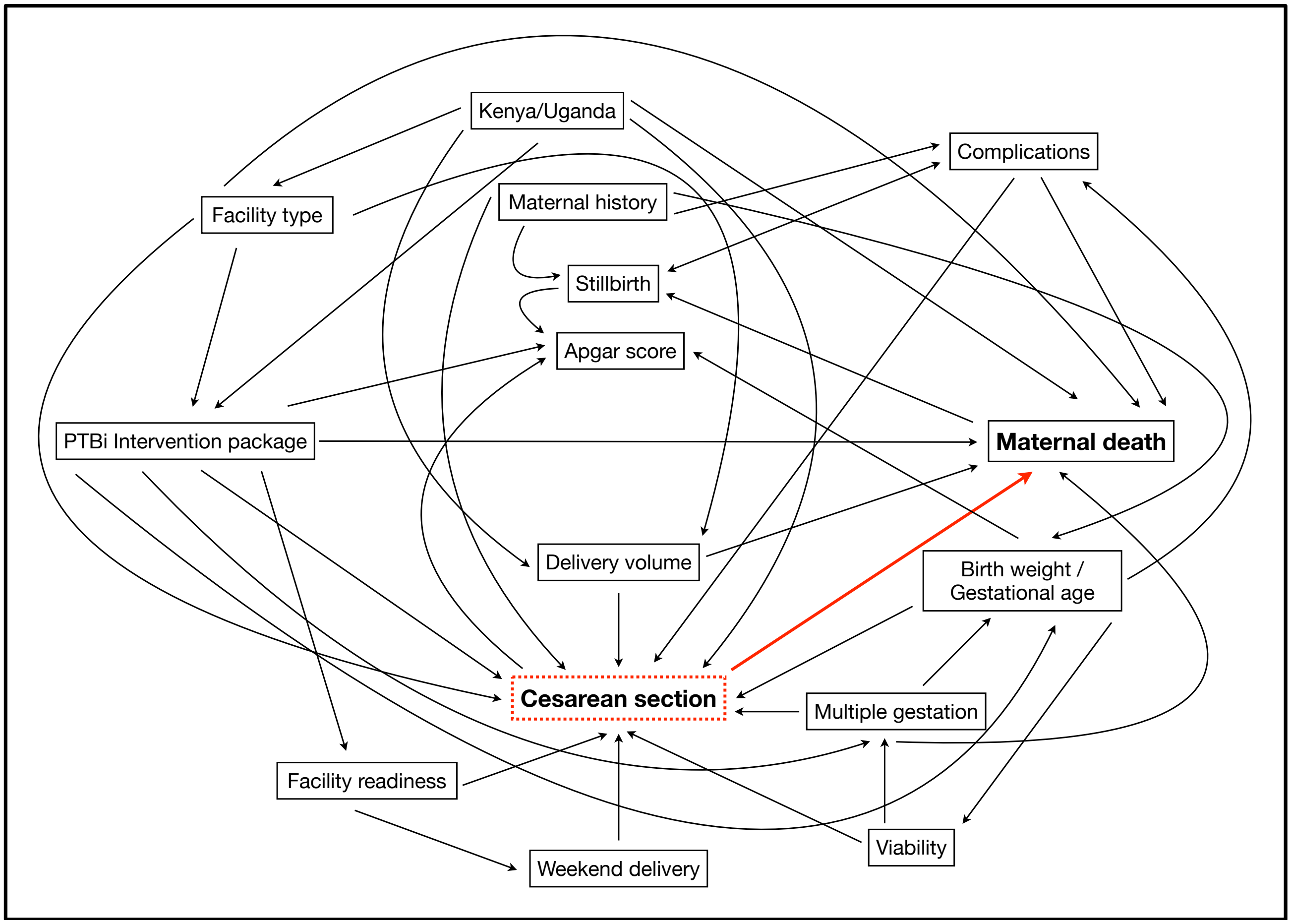

Table S1. Adjusted[!] odds ratios for types of stillbirths and cesarean sections.

	Vaginal Delivery % (n)	Cesarean Section % (n)	Adjusted Odds Ratio (95% CI)	P-value
**Antepartum/macerated stillbirth ***				
Live birth	75.7 (56,718)	24.3 (18,193)	1.00	
Yes	79.6 (1,200)	20.4 (308)	0.74 (0.65, 0.84)	<0.001
**Intrapartum/fresh stillbirth ***				
Live birth	75.7 (56,718)	24.3 (18,193)	1.00	
Yes	60.4 (1,084)	39.6 (710)	1.79 (1.63, 1.98)	<0.001

! Adjusted for birth weight, annual delivery volume, type of facility, country and exposure to PTBi intervention. The models are single-level logistic regression.
** Based on the neonatal dataset of 80,976*

Ghosh R, *et al. BMJ Open* 2022; 12:e055904. doi: 10.1136/bmjopen-2021-055904

Table S2. Associations between pregnancy related maternal death and cesarean section stratified by birth outcome and referral status.[!]

		**Maternal mortality** (C Section × Stillbirths, $P_{interaction}$ <0.001*)		**Maternal mortality** (C Section × Referral Status, $P_{interactions}$ = 0.26 and 0.80*)		
		**Livebirths**	**All stillbirths**	**Not referred**	**Referred**	**Missing**
	n	66,098	2,954	39,519	6,482	21,472
**C Section** **	No	1.00	1.00	1.00	1.00	1.00
	Yes	3.94 (2.84, 5.46)	1.70 (0.95, 3.03)	3.61 (1.56, 8.37)	2.04 (0.79, 5.27)	3.10 (2.06, 4.65)
	FDR Unadjusted p-value	<0.001	0.07	0.003	0.14	0.001
	FDR adj p-value	0.0003	0.09	0.006	0.16	0.002
**Number of events**						
**C Section**	No	32	37	28	8	31
	Yes	44	25	33	12	22

! Adjusted for birth weight, annual delivery volume, type of facility and country.
** Interaction between the two variables and the p-value for the interaction term. For Referral Status, two p-values are reported because the variable has 3 categories. After adjusting for multiple comparison using False Discovery Rate (FDR) method, the FDR unadjusted interaction p-values changed from <0.001 to 0.0003 for stillbirth and from 0.26 to 0.29 and 0.80 to 0.83 for referral status..*

Ghosh R, *et al. BMJ Open* 2022; 12:e055904. doi: 10.1136/bmjopen-2021-055904

Table S3. Associations between adverse neonatal outcomes and cesarean section stratified by referral status.[!]

	**Stillbirth** (C Section × Referral Status, $P_{interaction}$ = 0.026 and 0.046*)			**Apgar <7** (C Section × Referral Status, $P_{interaction}$ <0.001 and 0.69*)			**Pre-discharge neonatal mortality** (C Section × Referral Status, $P_{interaction}$ = 0.85 and 0.003*)		
	**Not referred**	**Referred**	**Missing**	**Not referred**	**Referred**	**Missing**	**Not referred**	**Referred**	**Missing**
n	42,813	7,007	27,625	35,075	6,218	22,651	43,349	7,110	27,625
**C Section**** No	1.00	1.00	1.00	1.00	1.00	1.00	1.00	1.00	1.00
Yes	1.22 (0.96, 1.54)	0.86 (0.77, 0.97)	1.74 (1.22, 2.46)	1.96 (1.55, 2.48)	1.24 (1.05, 1.47)	2.02 (1.41, 2.89)	1.61 (1.37, 1.90)	1.69 (1.11, 2.59)	2.31 (1.82, 2.93)
FDR Unadj pval	0.10	0.012	0.002	<0.001	0.01	<0.001	<0.001	0.014	<0.001
FDR adj p-value	0.12	0.019	0.005	0.0003	0.02	0.0003	0.003	0.021	0.003
No. of events									
C Section. No	1,068	337	879	682	293	974	353	87	234
Yes	445	241	332	471	303	413	193	108	124

! Adjusted for birth weight, annual delivery volume, type of facility and country.

** Interaction between the two variables and the p-values for the interaction terms. Two p-values are reported because the referral variable has 3 categories. After adjusting for multiple comparison using False Discovery Rate (FDR) method, the interaction p-values changed to 0.04 and 0.06 for stillbirth, 0.0003 and 0.74 for Apgar and 0.85 and 0.006 for predischarge mortality. These p-values should be compared to the ones in the very top row.*

Ghosh R, *et al. BMJ Open* 2022; 12:e055904. doi: 10.1136/bmjopen-2021-055904
