# Supplementary material for: Stillbirth, neonatal and maternal mortality among caesarean births in Kenya and Uganda: a register-based prospective cohort study
Source: BMJ Open. 2022 Apr 6;12(4):e055904. doi: 10.1136/bmjopen-2021-055904 (PMC8987792; doi:10.1136/bmjopen-2021-055904)
Supplement: Author's manuscript [file bmjopen-2021-055904.draft_revisions.pdf]

BMJ Open is committed to open peer review. As part of this commitment we make the peer review history of every article we publish publicly available.

When an article is published we post the peer reviewers' comments and the authors' responses online. We also post the versions of the paper that were used during peer review. These are the versions that the peer review comments apply to.

The versions of the paper that follow are the versions that were submitted during the peer review process. They are not the versions of record or the final published versions. They should not be cited or distributed as the published version of this manuscript.

BMJ Open is an open access journal and the full, final, typeset and author-corrected version of record of the manuscript is available on our site with no access controls, subscription charges or pay-per-view fees (http://bmjopen.bmj.com).

If you have any questions on BMJ Open's open peer review process please email info.bmjopen@bmj.com

BMJ Open

Stillbirth, neonatal, and maternal mortality among cesarean births: evidence from Kenya and Uganda

Journal:	*BMJ Open*
Manuscript ID	bmjopen-2021-055904
Article Type:	Original research
Date Submitted by the Author:	27-Jul-2021

Complete List of Authors:	Ghosh, Rakesh; University of California San Francisco, Institute for Global Health Sciences<br>Santos, Nicole; University of California San Francisco, Institute for Global Health Sciences<br>Wanyoro, Anthony; Kenyatta University, Department of OB/GYN<br>Kim, Eliana; University of California San Francisco<br>Butrick, Elizabeth ; University of California San Francisco<br>Waiswa, Peter; Makerere University, School of Public Health<br>Walker, Dilys; University of California San Francisco Department of Obstetrics Gynecology and Reproductive Sciences; University of California San Francisco Global Health Sciences

Keywords:	Epidemiology < TROPICAL MEDICINE, OBSTETRICS, Quality in health care < HEALTH SERVICES ADMINISTRATION & MANAGEMENT

SCHOLARONE™
Manuscripts

I, the Submitting Author has the right to grant and does grant on behalf of all authors of the Work (as defined in the below author licence), an exclusive licence and/or a non-exclusive licence for contributions from authors who are: i) UK Crown employees; ii) where BMJ has agreed a CC-BY licence shall apply, and/or iii) in accordance with the terms applicable for US Federal Government officers or employees acting as part of their official duties; on a worldwide, perpetual, irrevocable, royalty-free basis to BMJ Publishing Group Ltd ("BMJ") its licensees and where the relevant Journal is co-owned by BMJ to the co-owners of the Journal, to publish the Work in this journal and any other BMJ products and to exploit all rights, as set out in our licence.

The Submitting Author accepts and understands that any supply made under these terms is made by BMJ to the Submitting Author unless you are acting as an employee on behalf of your employer or a postgraduate student of an affiliated institution which is paying any applicable article publishing charge ("APC") for Open Access articles. Where the Submitting Author wishes to make the Work available on an Open Access basis (and intends to pay the relevant APC), the terms of reuse of such Open Access shall be governed by a Creative Commons licence – details of these licences and which Creative Commons licence will apply to this Work are set out in our licence referred to above.

Other than as permitted in any relevant BMJ Author's Self Archiving Policies, I confirm this Work has not been accepted for publication elsewhere, is not being considered for publication elsewhere and does not duplicate material already published. I confirm all authors consent to publication of this Work and authorise the granting of this licence.

For peer review only

Stillbirth, neonatal, and maternal mortality among cesarean births: evidence from Kenya and Uganda

Rakesh Ghosh[1*§], Nicole Santos[1*], Elizabeth Butrick[1], Anthony Wanyoro[2], Peter Waiswa[3,4], Eliana Kim[5], Dilys Walker[1,6]

1. University of California, San Francisco, Institute for Global Health Sciences, USA
2. Kenyatta University, Department of OB/GYN, Kenya
3. Makerere University, School of Public Health, Uganda
4. Department of Global Public Health, Karolinska Institutet, Sweden
5. University of California, San Francisco, School of Medicine, USA
6. University of California, San Francisco, Department of OB/GYN and Reproductive Sciences, USA

* These authors contributed equally to this work.
§ Corresponding author: Rakesh Ghosh, Institute for Global Health Sciences, University of California San Francisco, 550 16th St, San Francisco, CA, 94158, USA. Email: Rakesh.Ghosh@ucsf.edu

Word count – 3745 (main text only not including references)

For peer review only

ARTICLE SUMMARY

Strengths and limitations of the study

- Using data from over 80,000 births across 10 facilities in Kenya and Uganda with a range of cesarean section rates, we found women who underwent cesarean section had higher risk of intrapartum stillbirths, pre-discharge neonatal mortality, and facility-based pregnancy-related maternal death compared to women who delivered vaginally.
- We also found that one-third of antepartum stillbirths were delivered by cesarean section, and there was a higher association between cesarean section and maternal mortality for a livebirth than for a stillbirth.
- Our results indicate the association between cesarean section and stillbirth was differential by referral status with lower association for stillbirth among referred-in mothers who delivered by cesarean section.
- We lacked data on various maternal characteristics such as Robson criteria for cesarean section, antenatal care coverage, indication for cesarean section, peri- and post-operative complications, and cause of death.
- We also lacked data on system-related factors such as time between cesarean section decision and incision, access to anesthesia, and personnel conducting the cesarean section.

For peer review only

ABSTRACT (300 words max)

Introduction: Access to safe and timely cesarean section (CS) can avert many maternal and neonatal deaths. While evidence separately links CS with maternal mortality and with neonatal outcomes in different settings, no data, to our knowledge, describes the inter-relationship between maternal mortality and stillbirth among deliveries by CS.

Methods: In this prospective cohort study spanning ten hospitals in Kenya and Uganda, we investigated the risks for adverse outcomes, including maternal mortality, stillbirth and pre-discharge neonatal mortality, in CS compared to vaginal deliveries (VD). Individual birth data were extracted from maternity registers between October 2016 and April 2019. The association of CS with outcomes was quantified using random effects logistic regression after accounting for potential confounders and clustering of births within facilities. Interaction was assessed using product terms.

Results: There were a total of 77,242 livebirths and 3,734 stillbirths. Overall, 24% of deliveries were by CS with a range of 9% to 49% across facilities. The adjusted odds ratios for stillbirth, pre-discharge neonatal mortality and maternal mortality after a CS were 1.3 (95% CI: 1.1, 1.6), 1.9 (95% CI: 1.6, 2.2) and 3.3 (95% CI: 2.2, 4.9), respectively, compared to a VD. The association between maternal mortality and CS was 3.9 (95% CI: 2.8, 5.5) when the delivery was a livebirth and 1.7 (95% CI: 1.0, 3.0) when it was a stillbirth. Post-hoc analyses showed that mothers who received a CS had a lower risk of stillbirth if they were documented as a referral.

Conclusion: In this context, CS births were at higher risk for worse outcomes compared to VD. Better understanding of CS use and associated adverse outcomes within the mother-baby dyad is necessary to identify opportunities to improve quality of intrapartum care.

Funding: This work was supported by the East Africa Preterm Birth Initiative, which was funded by the Bill and Melinda Gates Foundation (OPP1107312).

Keywords: cesarean section; maternal mortality, neonatal mortality; stillbirth

For peer review only

42 **Clinical trial registration**: This is a secondary analysis of data from a trial registered with Clinical Trials.gov,

43 NCT03112018

44

For peer review only

For peer review only - http://bmjopen.bmj.com/site/about/guidelines.xhtml

INTRODUCTION

As facility-based births increase, access to and performance of cesarean section (CS) is also increasing in many low- and middle-income countries (LMICs). However, while global CS rates increased from 12% in 2000 to 21% in 2015, the increase has been only 5% to 6% in East and South Africa.[1] CS rates are projected to near 30% globally by 2030, though Sub-Saharan Africa is estimated to remain below 8%.[2]

Low CS rates may indicate unmet need and missed opportunity to prevent adverse outcomes.[3,4] Evidence from 16 Sub-Saharan African countries between 2008 and 2011 showed that an average CS rate of approximately 4% was associated with increased maternal, neonatal and infant mortality, which may have been averted by increasing access to cesarean delivery for medically indicated cases.[5] Additionally, it is well-documented that lack of trained personnel or access to safe anesthesia, blood supply and essential drugs, contribute to CS-related adverse outcomes in many LMICs.[5–7] A review of 196 studies from 67 LMICs showed that rates of maternal death and stillbirth were 8 and 57 per 1000 CS procedures, and were highest in sub-Saharan Africa, at 11 and 83, respectively.[8] Given that CS is usually indicated for a maternal or neonatal complication, it is not surprising that CS is associated with higher maternal and neonatal mortality. Access to timely and safe CS is critical to avert poor outcomes within the mother-baby dyad.

For peer review only

Increasing CS rates to recommended levels, especially in resource limited settings, may not always confer incremental benefit.[9–11] Overuse of CS in such settings introduce unnecessary risk for both mothers and newborns by diverting facility resources from the clinically indicated cases to low-risk pregnancies.[6,12]

While evidence separately links CS with maternal mortality and CS with neonatal outcomes in different settings, no data, to our knowledge, describes the inter-relationship between maternal mortality and stillbirth among births by CS. Generally, if intrauterine demise is identified prior to delivery, there are few indications to perform a CS due to increased maternal risks.[13] To better understand this interaction, we investigated the

For peer review only - http://bmjopen.bmj.com/site/about/guidelines.xhtml

characteristics of CS performed across 10 facilities in Kenya and Uganda. In Uganda, facility-based CS rates were 22–32% in general hospitals and 20–25% in referral hospitals between 2012 and 2016.[14] In Kenya, 2014 Demographic and Health Survey (DHS) data showed an overall 13% CS rate among institutional births, with notable disparities between rural and urban populations and across socioeconomic levels.[15] Data from the East Africa Preterm Birth Initiative (PTBi), a cluster-randomized trial (CRCT), provides a unique opportunity to investigate maternal and neonatal outcomes in the CS capable facilities in the two countries.[16,17] In this study we investigated the interaction of risks for adverse maternal and perinatal outcomes (stillbirth, pre-discharge neonatal and maternal mortality) among CS compared to vaginal deliveries (VD).

METHODS

Study design and setting

This analysis used maternity register data for deliveries between October 2016 and April 2019 from 10 CS-capable facilities in Busoga Region, East-Central Uganda and Migori County, Kenya that were involved in a pair-matched CRCT conducted by the East Africa Preterm Birth Initiative (trial registration: NCT03112018).[16,17] Four facilities were private not-for-profit mission hospitals and six were public hospitals with 620-2420 and 1200-6700 deliveries per year, respectively. Four of the 10 facilities that are part of this study were in the control arm and three were in the intervention arm of the CRCT. The three remaining hospitals were large referral hospitals that received the intervention but were not part of the CRCT given the inability to pair match them.

Busoga Region and Migori County have a predominantly rural population of approximately three million and one million people, respectively. Most women in the two respective regions deliver in a facility (77% and 53%), though regional neonatal mortality rates (27 and 19 per 1000 live births) and stillbirth rates (17 and 10 per 1000 pregnancies) remain high.[18,19] In Uganda, health centers (level III) offer basic emergency obstetric care and are

For peer review only

linked to a referring facility (either a health center IV or a hospital) for comprehensive emergency obstetric care. In Kenya, referral to the county referral hospital from lower-level facilities is standard, though women may seek services within the private sector or across county borders.

Data sources, eligibility criteria and variables of interest

Anonymized individual level birth data were extracted monthly from facility maternity registers by PTBi study staff. These routine data sources are completed by frontline health care workers. As part of the CRCT, all facilities received data strengthening, including reinforcement of key indicator definitions and continuous feedback of maternity register completion and accuracy for key variables (e.g. gestational age, birth weight, Apgar, discharge status).[20,21] There was no routine follow-up after discharge.

Data collected include maternal (age, multiplicity, referral status) and newborn (birth weight, gestational age at birth, five-minute Apgar, infant sex) characteristics and maternal and newborn status at discharge. Maternal length of hospital stay was calculated using delivery and discharge dates. Mode of delivery was documented as CS, normal vaginal, assisted vaginal, breech, and vacuum extraction. For this analysis, births were excluded if mode of delivery was missing, or documented as an abortion or vacuum aspiration. Babies born before arrival to the facility were also excluded.

The main outcomes were defined as: (1) stillbirths (birth at ≥28 weeks of gestation with no signs of life); (2) pre-discharge neonatal mortality (death of a liveborn baby before facility discharge); and (3) facility-based pregnancy-related maternal death.

Birth outcome was determined by triangulating data from delivery status, baby discharge status, and Apgar scores at one and five minutes. In Kenya, stillbirths were categorized into intrapartum stillbirth (intrauterine death of a fetus during labour or delivery; recorded as “fresh stillbirth”) or antepartum stillbirth (intrauterine death of a fetus before the onset of labour, where the fetus showed degenerative changes, recorded as

For peer review only

For peer review only - http://bmjopen.bmj.com/site/about/guidelines.xhtml

"macerated stillbirth").[22] If either the delivery status or the baby discharge status were recorded as antepartum stillbirth or intrapartum stillbirth and the Apgar scores at one *and* five minutes were zero, their birth outcome was designated as originally documented; if the recording was stillbirth *and* all Apgar scores were zero, they were categorized as an undefined stillbirth. The Uganda maternity register did not differentiate between antepartum and intrapartum stillbirths and were categorized as stillbirths of undefined type. To reconcile the heterogeneity in documentation between countries, we included intrapartum, antepartum and undefined stillbirths into one overall group as stillbirths.

Statistical analysis

The association of CS with maternal and perinatal outcomes was quantified using logistic regression and results are presented as odds ratios (OR) with 95% confidence intervals (95% CI). The data were two-tiered – individual deliveries clustered within facilities. We used mixed effect models with a random intercept for each facility to account for the non-independence of the deliveries within facilities. When stratified by types of stillbirth, the number of outcomes reduced, and the models did not converge. Hence, we fit single-level logistic regression to gain insight into how the associations differ by stillbirth sub-types (supplemental results only).

To specify a parsimonious model, we used a directed acyclic graph (DAG)[23] and change-in-estimate confounder selection method.[24] The DAG identified a necessary and sufficient set of potential confounders that required adjustment. We then used the change-in-estimate method of statistical modelling to identify the final set of potential confounders, with 5% change in the exposure-outcome association as the criterion. More explanation about the statistical modelling and the directed paths are presented in Figure S1.

Interaction was investigated using a product term in the models. If the product term was significant at the 5% significance level, the models were stratified by the interacting variable. Led by the descriptive results on referrals, we framed a post-hoc objective to examine interaction between CS and referral status on adverse

For peer review only - http://bmjopen.bmj.com/site/about/guidelines.xhtml

outcomes. To minimize chances of false-positive associations, we adjusted for multiple comparisons using false discovery rate.[25] The analysis was conducted in STATA 16.1.

Ethical considerations

The PTBi EA CRCT was granted ethical approvals from Higher Degrees, Research and Ethics Committee (MUSPH HDREC 395) from Makerere University; KEMRI Scientific and Ethics Review Committee (KEMRI/SERU/CCR/0034/3251) and UCSF Committee on Human Research (16-19162). Permission to extract non-identifiable data from maternity registers was allowed under these approvals.

Patient and public involvement

Our research question was informed by an observation that intrapartum stillbirth rates were high, especially among women who had CS. Since undergoing a CS involves added risk and a longer recovery period, we expect that women are more likely to consent to a CS if they anticipate a greater chance of an improved outcome. Given CS is often done for complications that lead to poorer outcomes, we decided to assess CS and maternal and perinatal outcomes in this context. We abstracted delivery and newborn care data for all women who delivered during the study period from the facility maternity register, under an IRB-approved waiver of consent. As this was a secondary analysis of a CRCT, all women who gave birth at a given facility, received the same standard of care. Patients were not involved in the design or conduct of the study, other than being a participant. Study results were shared with participating facilities' administration and staff by our in-country teams. However, results will not be disseminated directly to women.

We appreciate all the facility healthcare workers, leadership and administration who made this work possible. We thank all members of the PTBi Kenya and Uganda Implementation Research Collaborative, particularly the data teams who helped strengthen and collect data from facility maternity registers. In

For peer review only

For peer review only - http://bmjopen.bmj.com/site/about/guidelines.xhtml

particular, we convey our heartfelt thanks to the study participants for their participation and immensely valuable contribution to this study.

RESULTS

The final analytical dataset is shown in Figure 1. PTBi EA collected information on 83,867 deliveries across the 10 CS capable facilities, of which 80,976 had birth outcomes. To avoid double counting for multiple gestation pregnancies, we considered multiples as a single delivery event (n=78,451) for maternal outcomes.

There were 164 maternal deaths, 3,734 stillbirths and 77,242 live births. The maternal mortality ratio was 212 per 100,000 livebirths; the stillbirth rate was 46 per 1,000 births; and the pre-discharge neonatal mortality rate was 16 per 1,000 livebirths. Of the total stillbirths, 374 (10%) were documented as intrapartum stillbirths, 353 (9%) as antepartum stillbirths in Kenya, and 3,007 (81%) were stillbirths of undefined type in Uganda. The Ugandan facilities did not record type of stillbirth.

Overall, 24% of deliveries were by CS (19,155/78,451) with a range of 9% to 49% across facilities. Table 1 shows that private not-for-profit mission facilities performed more CS (38%) than public facilities (21%). Neither exposure to the PTBi intervention nor annual delivery volume were significantly associated with CS rates.

Maternal and neonatal characteristics by mode of delivery are shown in Table 1. There was an increased association between CS and referred-in status, compared to those not referred. The length of hospital stay after delivery was significantly associated with mode of delivery with about two-thirds of mothers who were hospitalized for seven days or longer having undergone a CS (OR 1.9, 95% CI: 1.8, 2.0). Overall, about 6% of women who underwent a CS had a hospital stay of more than seven days compared to <1% for vaginal delivery. The median (interquartile range) length of stay was 3 (1, 5) days for a CS, compared to 1 (0, 1) day for a VD. Over a third of the women (36%) who had multiple gestations underwent CS, with an OR of 1.8 (95% CI: 1.5, 2.0), relative to singletons.

For peer review only

For peer review only - http://bmjopen.bmj.com/site/about/guidelines.xhtml

For neonatal characteristics, relative to infants with gestational age (GA) between 37 and 40 weeks, those who were <32 completed weeks at birth had reduced odds for CS, while those between 32 and <37 weeks and >40 weeks had increased odds for CS (Table 1). Likewise, relative to infants between 2500 and 3999 grams, those weighing <1000 grams had reduced odds of CS, while those between 1000 and 2499 grams and those ≥4000 grams had increased odds of CS. There was an increased association between CS and Apgar <7 at five minutes (OR 1.8, 95% CI: 1.3, 2.4), compared to those with an Apgar score ≥7.

Table 2 presents the adjusted OR (aOR) for the association between CS and adverse maternal or perinatal health outcomes. The aORs were 1.3 (95% CI: 1.1, 1.6) for stillbirth and 1.9 (95% CI: 1.6, 2.2) for pre-discharge neonatal mortality when the mode of delivery was CS compared to VD. The aOR for maternal death after a CS was 3.3 times higher (95% CI: 2.2, 4.9) compared to VD. Table S1 presents CS associations by stillbirth types, which is decreased for antepartum stillbirth (0.4, 95% CI: 0.3, 0.6) and increased for intrapartum stillbirth (1.4, 95% CI: 1.1, 1.7).

Figure 2 shows that the risk for maternal death after CS was higher for a livebirth than for a stillbirth. For livebirths, the aOR for maternal death after CS was 3.9 (95% CI: 2.8, 5.5), compared to VD, while it was 1.7 (95% CI: 1.0, 3.0) for stillbirths. For maternal mortality after CS, the association did not statistically differ by referral status of the mothers. Numerical estimates are presented in Table S2.

Figure 3 shows that when the mother was not referred, the aOR for stillbirth at CS was 1.2 (95% CI: 1.0, 1.5), compared to a VD and 0.9 (95% CI: 0.8, 1.0) when the delivery was referred-in. For pre-discharge neonatal mortality, the association did not statistically differ by referral status of the mothers. Table S3 presents numerical estimates for Figure 3.

For peer review only

Apgar score <7 at five minutes is an indicator of neonatal well-being, as well as success of resuscitation. Table S4 shows that when the mother was not referred, the odds for Apgar <7 after CS was 2.0 (95% CI: 1.6, 2.5), compared to a VD and 1.2 (95% CI: 1.1, 1.5) when the delivery was referred-in.

The statistical significance for the associations presented in Table 2 as well as the interactions remained largely unchanged after adjustment for multiple comparisons.

DISCUSSION

This analysis demonstrates an increased association of CS with adverse maternal and perinatal outcomes in two sub-Saharan countries. Compared to women who had a VD, those who underwent CS had increased association with maternal death, stillbirth and pre-discharge neonatal mortality. We found a higher association between CS and maternal mortality for a livebirth than for a stillbirth. This novel differential association by birth outcome is subject to independent verification but underscores the need to approach the clinical situation and the decision-making from the lens of the mother-baby dyad, that optimizes improved outcomes. We also found that women who were documented as referred-in and underwent a CS had a lower risk of stillbirth than those who were not referred-in and underwent a CS. While the risk for maternal mortality after a CS was not statistically different by referral status, it generally trended towards lower risk of poor outcome if documented as referred-in.

Our overall finding that women who had a CS had higher risk of maternal death is consistent with previous studies and not surprising given the indications for CS include high-risk conditions for both mothers and newborns. In a South African study, CS rates at various facilities ranged between 15% and 28% and the risk of maternal death after CS was 2.8 times that of VD.[26] A study across 41 referral hospitals in Mali and Senegal demonstrated that women who underwent intrapartum CS were at higher risk for maternal mortality compared to women who had a spontaneous VD, as well as pre-discharge neonatal mortality.[27]

For peer review only - http://bmjopen.bmj.com/site/about/guidelines.xhtml

Our findings suggest the risk of maternal death after a CS was higher for a livebirth than it was for a stillbirth. With limited relevant data in the registries and no access to maternal death reviews, we could not ascertain the cause and time of death, associated complications, and time between decision to incision, limiting the ability to interpret these results. At Mulago National Referral Hospital in Uganda, shorter decision-to-CS delivery times were associated with fetal distress, while longer times were associated with pre-eclampsia and premature rupture of membranes (PROM), hinting towards increased attention and action to ensure fetal well-being.[28] This could be because mothers who have pre-eclampsia or PROM may require stabilization or antenatal corticosteroids prior to CS, unlike a mother who is diagnosed with non-reassuring fetal heart tones. However, elevated odds of maternal death even with stillbirth (but less than livebirth) bring to light the need for careful evaluation on indications for cesarean and quality of cesarean care.

We found higher odds of stillbirth among women who received a CS compared to VD. Studies have linked unmet need for CS to increased intrapartum stillbirths.[4,29] In this study, more than 30% of stillbirths were delivered by CS suggesting that intrapartum quality of care and CS timeliness may contribute to stillbirth outcomes. About 14% of all antepartum stillbirths were delivered by CS but this data is from Kenya only and warrants cautious interpretation. Nonetheless, it raises questions of adequate fetal monitoring prior to CS initiation, appropriate indication for CS, and timeliness. It is unclear whether these were conducted for clinically appropriate indications, such as uterine rupture, prior CS, or if there was a lack of alternative capacity to manage stillbirth. This paucity of data highlights the value of auditing CS cases particularly those in which a stillbirth is delivered. This may reveal important and urgent opportunities for quality improvement of intrapartum care in these settings.

Increased CS rates among referred-in mothers could contribute to higher rates of stillbirths[30,31] and maternal deaths,[26] due to delays in transportation, timeliness, etc. However, we were intrigued to note that referred-in mothers had a lower risk of stillbirth and maternal death, though the latter was not significant. We also found

For peer review only - http://bmjopen.bmj.com/site/about/guidelines.xhtml

that compromised newborn well-being at birth (Apgar score <7 at five minutes) showed a similar relationship, suggesting that referred-in deliveries may have been exposed to more timely intervention or increased evidence-based practices during the intrapartum period, such as newborn resuscitation. While the underlying contributors to this finding are unclear; some hypotheses might be: (1) referred-in women were stabilized prior to arrival; (2) prior assessment led to more timely CS upon arrival to the receiving facility; (3) referred-in women had more time to mobilize resources for a timely CS; and (4) decision to refer was made before the onset of severe complications (e.g. early diagnoses of CS indication) or due to non-medical reasons. Missingness of the referral variable demands cautious interpretation. However, the intriguing results suggest that the existing referral system may be functional in identifying and managing high-risk women and their newborns. At the least, this finding deserves further exploration in this particular referral system and in similar contexts for a deeper understanding.

Collectively, these findings highlight the need to assess gaps in CS quality of care and system bottlenecks to improve maternal and perinatal outcomes. CS-specific checklists and audits have improved uptake of evidence-based practices, clinical documentation and quality of care.[32,33] Few vacuum or forceps deliveries were recorded in maternity registers, which should be optimized to avoid unnecessary CS, particularly among known intrauterine fetal deaths.[34,35] Another obvious area of improvement is documentation of clinical diagnoses, indications for CS, and referral status. In the absence of robust data for clinical indications for CS, we cannot rule out elective CS, though clinically indicated CS likely constitute a vast majority of all CS in this context.[36] While PTBi EA invested in data strengthening across the study facilities, this largely focused on variables such as gestational age, birth weight, Apgar and discharge status.[16] Maternity registers use free text to document final diagnosis, delivery complications and referral status. In this study, among women who had CS and stillbirth, 40% had no diagnosis documented; 63% and 43% of women who died after a VD or a CS had no diagnosis, respectively. Due to these data issues, we cannot assess whether conditions such as pre- or peri-operative obstetric hemorrhage, hypertensive disorders, infection or anesthesia complications may have contributed to

For peer review only - http://bmjopen.bmj.com/site/about/guidelines.xhtml

deaths, all of which have been independently associated with maternal mortality.[37] Given the relatively high CS rates in some of the facilities and the rising rates globally, inclusion of CS indications may be warranted in birth registries.

Likewise, differentiation between intrapartum and antepartum stillbirths would undoubtedly inform opportunities and curb the potential overuse of CS for non-indications, particularly in cases of known stillbirth. Overall, rate of intrapartum stillbirths has previously been suggested as a maternal quality care indicator by WHO and other stakeholders.[38] Disaggregating this indicator by mode of delivery could serve as a quality of intrapartum care indicator, particularly in the context of rising CS rates across LMICs[39] and the fact that CS should be considered a last resort for delivering a stillborn infant due to the increased risk to the mother.[13]

Strengths and Limitations

The study has several limitations. Outcomes were ascertained by facility personnel and there may be data quality issues as maternity registers are often completed in the context of high workload. For example, approximately 40 cases had conflicting delivery and discharge status, and were excluded. Second, we lacked robust data on various maternal characteristics such as Robson criteria for CS, antenatal care coverage, indication for CS, peri- and post-operative complications, and cause of death. We also lacked data on system-related factors such as time between CS decision and incision, access to anesthesia, and personnel conducting the CS. As noted above, this information would have helped contextualize our findings.

Regardless of these limitations, we used robust design and analytic approaches to quantify associations and have been cautious in drawing conclusions. By leveraging existing data systems, we captured real-world contexts and underscore the importance of data standardization, completeness and accuracy. Another strength of this study was the availability of pregnancy-related mortality data across heterogeneous types of facilities in two countries. Given the duration of the PTBi EA program, we had sufficient sample size to power the investigation

For peer review only - http://bmjopen.bmj.com/site/about/guidelines.xhtml

for maternal mortality and investigate a novel interaction between CS and birth outcome. The results are unlikely to be chance findings, given the significance level after adjustment for multiple comparisons.

CONCLUSION

In this study, CS births were at higher risk for worse maternal and perinatal outcomes compared to VD. Additionally, the risk of maternal death after a CS was higher among livebirths than it was for stillbirths. Better understanding of CS use and associated adverse outcomes within the mother-baby dyad is necessary to identify opportunities to improve quality of intrapartum care. Detailed case documentation including indications for CS, referral and causes of death is needed to shed light on these issues and identify pathways to improve care.

For peer review only

For peer review only - http://bmjopen.bmj.com/site/about/guidelines.xhtml

DECLARATIONS

Acknowledgements
We appreciate all the facility healthcare workers, leadership and administration who made this work possible. We thank all members of the Preterm Birth Initiative Kenya & Uganda Implementation Research Collaborative, particularly the data teams who helped strengthen and collect data from facility maternity registers. In particular, we convey our heartfelt thanks to the study participants for their participation and immensely valuable contribution to this study.

Author contributions
DW, EB, NS and RG contributed to study conceptualization. RG led data analysis. RG and NS drafted the manuscript. All authors contributed to data synthesis and interpretation. All authors reviewed and approved the final version of the manuscript.

Details of Ethics Approval
The East Africa Preterm Birth Initiative was granted ethical approvals from Higher Degrees, Research and Ethics Committee (MUSPH HDREC 395) from Makerere University; KEMRI Scientific and Ethics Review Committee (KEMRI/SERU/CCR/0034/3251) and UCSF Committee on Human Research (16-19162). Permission to extract non-identifiable data from maternity registers was allowed under these approvals.

Funding
This work was supported by the East Africa Preterm Birth Initiative, which was funded by the Bill and Melinda Gates Foundation (OPP1107312). The funders did not have input on analysis or interpretation of results.

Disclosure of interests
All authors declare no conflict of interest.

Data sharing statement
Anonymized data will be made available upon reasonable request to: Dilys.Walker@ucsf.edu

view only

For peer review only - http://bmjopen.bmj.com/site/about/guidelines.xhtml

REFERENCES

1. Boerma T, Ronsmans C, Melesse DY, Barros AJD, Barros FC, Juan L, et al. Global epidemiology of use of and disparities in caesarean sections. Lancet [Internet]. 2018;392(10155):1341–8. Available from: http://dx.doi.org/10.1016/S0140-6736(18)31928-7

2. Betran AP, Ye J, Moller B, Souza JP. Trends and projections of caesarean section rates : global and regional estimates. 2021;1–8.

3. Belizán JM, Minckas N, McClure EM, Saleem S, Moore JL, Goudar SS, et al. An approach to identify a minimum and rational proportion of caesarean sections in resource-poor settings: a global network study. Lancet Glob Heal. 2018;6(8):e894–901.

4. Harrison MS, Pasha O, Saleem S, Ali S, Chomba E, Carlo WA, et al. A prospective study of maternal, fetal, and neonatal outcomes in the setting of cesarean section in low- and middle-income countries. Acta Obs Gynecol Scand. 2018;96(4):410–20.

5. Irani M, Deering S. Challenges affecting access to cesarean delivery and strategies to overcome them in low-income countries. Int J Gynecol Obstet. 2015;131(1):30–4.

6. Harrison MS, Goldenberg RL. Cesarean section in sub-Saharan Africa. Matern Heal Neonatol Perinatol [Internet]. 2016;2(1):1–10. Available from: http://dx.doi.org/10.1186/s40748-016-0033-x

7. Epiu I, Wabule A, Kambugu A, Mayanja-Kizza H, Tindimwebwa JVB, Dubowitz G. Key bottlenecks to the provision of safe obstetric anaesthesia in low- income countries; a cross-sectional survey of 64 hospitals in Uganda. BMC Pregnancy Childbirth. 2017;17(1):1–8.

8. Sobhy S, Arroyo-Manzano D, Murugesu N, Karthikeyan G, Kumar V, Kaur I, et al. Maternal and perinatal mortality and complications associated with caesarean section in low-income and middle-income countries: a systematic review and meta-analysis. Lancet [Internet]. 2019;393(10184):1973–82. Available from: http://dx.doi.org/10.1016/S0140-6736(18)32386-9

9. Betrán AP, Temmerman M, Kingdon C, Mohiddin A, Opiyo N, Torloni MR, et al. Interventions to reduce unnecessary caesarean sections in healthy women and babies. Lancet [Internet]. 2018;392(10155):1358–68. Available from: http://dx.doi.org/10.1016/S0140-6736(18)31927-5

10. WHO. WHO Statement on Caesarean Section Rates [Internet]. 2015. Available from: https://www.who.int/reproductivehealth/publications/maternal_perinatal_health/cs-statement/en/

11. Ye J, Zhang J, Mikolajczyk R, Torloni MR, Gülmezoglu AM, Betran AP. Association between rates of caesarean section and maternal and neonatal mortality in the 21st century: A worldwide population-based ecological study with longitudinal data. BJOG An Int J Obstet Gynaecol. 2016;123(5):745–53.

12. Litorp H, Kidanto HL, Nystrom L, Darj E, Essén B. Increasing caesarean section rates among low-risk groups: A panel study classifying deliveries according to Robson at a university hospital in Tanzania. BMC Pregnancy Childbirth. 2013;13:1–10.

13. Chakhtoura NA, Reddy UM. Management of stillbirth delivery. Semin Perinatol [Internet]. 2015;39(6):501–4. Available from: http://dx.doi.org/10.1053/j.semperi.2015.07.016

14. Atuheire EB, Opio DN, Kadobera D, Ario AR, Matovu JKB, Harris J, et al. Spatial and temporal trends of cesarean deliveries in Uganda: 2012-2016. BMC Pregnancy Childbirth. 2019;19(1):1–8.

15. Ochieng Arunda M, Agardh A, Asamoah BO. Cesarean delivery and associated socioeconomic factors and

For peer review only - http://bmjopen.bmj.com/site/about/guidelines.xhtml

neonatal survival outcome in Kenya and Tanzania: analysis of national survey data. Glob Health Action [Internet]. 2020;13(1). Available from: https://doi.org/10.1080/16549716.2020.1748403

16. Walker D, Otieno P, Butrick E, Namazzi G, Achola K, Merai R, et al. Effect of a quality improvement package for intrapartum and immediate newborn care on fresh stillbirth and neonatal mortality among preterm and low-birthweight babies in Kenya and Uganda: a cluster-randomised facility-based trial. Lancet Glob Heal [Internet]. 2020;8(8):e1061–70. Available from: http://dx.doi.org/10.1016/S2214-109X(20)30232-1

17. Otieno P, Waiswa P, Butrick E, Namazzi G, Achola K, Santos N, et al. Strengthening intrapartum and immediate newborn care to reduce morbidity and mortality of preterm infants born in health facilities in Migori County, Kenya and Busoga Region, Uganda: A study protocol for a randomized controlled trial. Trials. 2018;19(1).

18. Kenya Demographic and Health Survey 2015. Nairobi, Kenya and Rockville, Maryland, USA; 2015.

19. Uganda Demographic and Health Survey 2016. Kampala, Uganda and Rockville, Maryland, USA; 2018.

20. Keating R, Merai R, Mubiri P, Kajjo D, Otare C, Mugume D, et al. Assessing effects of a data quality strengthening campaign on completeness of key fields in facility-based maternity registers in Kenya and Uganda. East Africa J Appl Heal Monit Eval. 2019;1–7.

21. Waiswa P, Higgins B V., Mubiri P, Kirumbi L, Butrick E, Merai R, et al. Pregnancy outcomes in facility deliveries in Kenya and Uganda: A large cross-sectional analysis of maternity registers illuminating opportunities for mortality prevention. PLoS One [Internet]. 2020;15(6):1–17. Available from: http://dx.doi.org/10.1371/journal.pone.0233845

22. Lawn JE, Blencowe H, Waiswa P, Amouzou A, Mathers C, Hogan D, et al. Stillbirths: rates, risk factors, and acceleration towards 2030. Lancet. 2016 Feb;387(10018):587–603.

23. Greenland S, Pearl J, Robins JM. Causal diagrams for epidemiologic research. Vol. 10, Epidemiology. 1999. p. 37–48.

24. Analysis E. Commentary Modeling and Variable Selection in Epidemiologic Analysis SANDER GREENLAND, MS, DrPH. 1989;79(3):1–21.

25. Storey JD. The positive false discovery rate: A Bayesian interpretation and the q-value. Ann Stat. 2003;31(6):2013–35.

26. Gebhardt GS, Fawcus S, Moodley J, Farina Z. Maternal death and caesarean section in South Africa: Results from the 2011 - 2013 saving mothers report of the national committee for confidential enquiries into maternal deaths. South African Med J. 2015;105(4):287–91.

27. Briand V, Dumont A, Abrahamowicz M, Sow A, Traore M, Rozenberg P, et al. Maternal and Perinatal Outcomes by Mode of Delivery in Senegal and Mali: A Cross-Sectional Epidemiological Survey. PLoS One. 2012;7(10):6–13.

28. Hughes NJ, Namagembe I, Nakimuli A, Sekikubo M, Moffett A, Patient CJ, et al. Decision-to-delivery interval of emergency cesarean section in Uganda: A retrospective cohort study. BMC Pregnancy Childbirth. 2020;20(1):1–10.

29. Goldenberg RL, McClure EM, Bann CM. The relationship of intrapartum and antepartum stillbirth rates to measures of obstetric care in developed and developing countries. Acta Obstet Gynecol Scand.

For peer review only

For peer review only - http://bmjopen.bmj.com/site/about/guidelines.xhtml

2007;86(11):1303–9.

30. van Duinen AJ, Westendorp J, Kamara MM, Forna F, Hagander L, Rijken MJ, et al. Perinatal outcomes of cesarean deliveries in Sierra Leone: A prospective multicenter observational study. Int J Gynecol Obstet. 2020;150(2):213–21.

31. Okonofua FE, Ntoimo LFC, Ogu R, Galadanci H, Mohammed G, Adetoye D, et al. Prevalence and determinants of stillbirth in Nigerian referral hospitals: A multicentre study. BMC Pregnancy Childbirth. 2019;19(1):1–9.

32. Dekker L, Houtzager T, Kilume O, Horogo J, van Roosmalen J, Nyamtema AS. Caesarean section audit to improve quality of care in a rural referral hospital in Tanzania. BMC Pregnancy Childbirth. 2018;18(1):1–7.

33. Sun M, Patauli D, Bernstein PS, Goffman D, Nathan LM. Use of a cesarean delivery checklist in an African maternity ward to improve management and reduce length of hospital stay. Int J Gynecol Obstet. 2020;(January):1–6.

34. Nolens B, Namiiro F, Lule J, van den Akker T, van Roosmalen J, Byamugisha J. Prospective cohort study comparing outcomes between vacuum extraction and second-stage cesarean delivery at a Ugandan tertiary referral hospital. Int J Gynecol Obstet. 2018;142(1):28–36.

35. Litorp H, Kidanto HL, Rööst M, Abeid M, Nyström L, Essén B. Maternal near-miss and death and their association with caesarean section complications: A cross-sectional study at a university hospital and a regional hospital in Tanzania. BMC Pregnancy Childbirth. 2014;14(1):1–10.

36. Chu K, Cortier H, Maldonado F, Mashant T, Ford N, Trelles M. Cesarean Section Rates and Indications in Sub-Saharan Africa: A Multi-Country Study from Medecins sans Frontieres. PLoS One. 2012;7(9):5–10.

37. Bishop D, Dyer RA, Maswime S, Rodseth RN, van Dyk D, Kluyts HL, et al. Maternal and neonatal outcomes after caesarean delivery in the African Surgical Outcomes Study: a 7-day prospective observational cohort study. Lancet Glob Heal. 2019;7(4):e513–22.

38. Madaj B, Smith H, Mathai M, Roos N, Van Den Broek N. Developing global indicators for quality of maternal and newborn care: A feasibility assessment. Bull World Health Organ. 2017;95(6):445-452I.

39. Boatin AA, Schlotheuber A, Betran AP, Moller AB, Barros AJD, Boerma T, et al. Within country inequalities in caesarean section rates: Observational study of 72 low and middle income countries. BMJ. 2018;360.

For peer review only

For peer review only - http://bmjopen.bmj.com/site/about/guidelines.xhtml

FIGURES CAPTION LIST

Figure 1: Flow diagram describing the final analytic samples and exclusions.

Figure 2: *Adjusted odds ratios (aOR and 95% Confidence Interval) for facility-based pregnancy related death and cesarean section stratified by birth outcome and referral status.* [!]
! Adjusted for birth weight, annual delivery volume, type of facility, country and exposure to PTBi intervention. Note – After adjusting for multiple comparison using False Discovery Rate method, the interaction p-values changed to 0.0003 and 0.29, respectively.

Figure 3: Adjusted odds ratios (aOR and 95% Confidence Interval) for adverse neonatal outcomes and cesarean section stratified by referral status. [!]
! Adjusted for birth weight, annual delivery volume, type of facility, country and exposure to PTBi intervention. Note – After adjusting for multiple comparison using False Discovery Rate method, the interaction p-values changed to 0.04 for stillbirth and remained unchanged at 0.85 for predischarge mortality.

For peer review only

Table 1. Facility, maternal and neonatal characteristics for vaginal deliveries and cesarean sections.

FACILITY CHARACTERISTICS	Vaginal Delivery % (n)	Cesarean Section % (n)	Odds Ratio (95% CI)	p-value (trend)
**Facility type***				
Public	78.7 (50,049)	21.3 (13,572)	1.00	<0.001
Private	62.4 (9,247)	37.7 (5,583)	3.07 (1.71, 5.51)	
**Exposure to PTBi intervention***				
No	75.7 (50,378)	24.4 (16,218)	1.00	0.455
Yes	75.3 (8,918)	24.8 (2,937)	1.51 (0.51, 4.46)	
**Annual delivery volume***				
< 1000 (Low)	68.4 (918)	31.7 (425)	1.46 (0.65, 3.28)	0.594
1000 – < 2800 (Medium)	73.0 (16,890)	27.0 (6,251)	1.00	
≥ 2800 (High)	76.9 (41,488)	23.1 (12,479)	0.76 (0.28, 2.04)	

MATERNAL & NEONATAL CHARACTERISTICS	Vaginal Delivery % (n)	Cesarean Section % (n)	Odds Ratio (95% CI)	p-value
**Maternal age (years)***				
< 18	76.4 (3,788)	23.6 (1,168)	1.06 (0.90, 1.26)	0.823
18 – ≤ 35	75.7 (51,924)	24.3 (16,686)	1.00	
36 – 53	74.0 (3,151)	26.1 (1,110)	1.04 (0.93, 1.16)	
**Delivery referred in***				
No	75.5 (32,823)	24.5 (10,661)	1.00	0.17
Yes	55.0 (3,847)	45.0 (3,146)	2.33 (2.06, 2.64)	
Missing	80.9 (22,542)	19.1 (5,324)	0.98 (0.83, 1.15)	
**Day of delivery***				
Monday – Friday	75.2 (41,781)	24.7 (13,773)	1.00	0.009
Weekend	76.7 (16,735)	23.3 (5,086)	0.93 (0.88, 0.98)	
**Maternal length of stay ***				
< 7 days	76.7 (57,593)	23.3 (17,462)	1.00	<0.001
≥ 7 days	33.4 (543)	66.6 (1,083)	1.93 (1.82, 2.04)	
**Multiple gestation***				
Singleton	75.9 (57,717)	24.1 (18,286)	1.00	<0.001
Twins, triplets or quadruplets	64.5 (1,579)	35.5 (869)	1.77 (1.53, 2.04)	
**Preterm birth (<37 completes weeks)***				
No	75.9 (52,493)	24.1 (16,708)	1.00	0.016
Yes	73.6 (6,803)	26.5 (2,447)	1.08 (1.01, 1.15)	
**Gestational age (weeks)***				
20 – < 28	85.8 (338)	14.2 (56)	0.57 (0.41, 0.80)	<0.001
28 – < 32	80.8 (1,337)	19.2 (318)	0.74 (0.60, 0.91)	
32 – < 37	74.1 (5,170)	25.9 (1,809)	1.14 (1.03, 1.25)	
37 – < 40	75.7 (34,303)	24.3 (10,986)	1.00	
≥ 40	76.2 (8,811)	23.8 (2,754)	1.08 (1.01, 1.15)	
**Low birth weight (< 2500 grams)****				
No	75.9 (52,819)	24.1 (16,777)	1.00	<0.001
Yes	71.1 (5,926)	28.9 (2,405)	1.22 (1.12, 1.33)	
**Birth weight (grams)****				
< 1000	89.9 (257)	10.1 (29)	0.34 (0.18, 0.66)	<0.001
1000 – 2499	71.1 (5,926)	28.9 (2,405)	1.24 (1.14, 1.35)	
2500 – 3999	76.2 (50,006)	23.8 (15,599)	1.00	
≥ 4000	70.5 (2,813)	29.5 (1,178)	1.39 (1.25, 1.55)	
**Apgar score at 5 minutes ****				
≥ 7	75.7 (47,154)	24.3 (15,159)	1.00	<0.001
< 7	63.0 (2,135)	37.0 (1,255)	1.78 (1.31,2.40)	

** Based on the delivery dataset of 78,451 mothers; ** Based on the neonatal dataset of 80,976*

For peer review only

For peer review only - http://bmjopen.bmj.com/site/about/guidelines.xhtml

Table 2. Adjusted[!] odds ratios for neonatal and maternal outcomes amongst those who had cesarean sections.

	Vaginal Delivery % (n)	Cesarean Section % (n)	Adjusted Odds Ratio (95% CI)	P-value	Adjusted P-value[@]
**Stillbirth ***					
No	75.5 (58,338)	24.5 (18,904)	1.00	0.006	0.01
Yes	69.0 (2,576)	31.0 (1,158)	1.33 (1.09, 1.63)		
**Pre-discharge neonatal mortality ***					
No	75.4 (60,174)	24.6 (19,606)	1.00	<0.001	0.0003
Yes	61.9 (740)	38.1 (456)	1.88 (1.64, 2.16)		
**Maternal mortality ****					
No	75.2 (52,694)	24.8 (17,419)	1.00	<0.001	0.0003
Yes	49.4 (77)	50.6 (79)	3.28 (2.21, 4.88)		

! Adjusted for birth weight, annual delivery volume, type of facility and country and exposure to PTBi intervention.

[@]P-values adjusted for multiple comparison using False Discovery Rate method.

** Based on the neonatal dataset of 80,976*

*** Based on the delivery dataset of 78,451 mothers*

For peer review only

Figure 1. Flow diagram describing the final analytic samples and the exclusions.

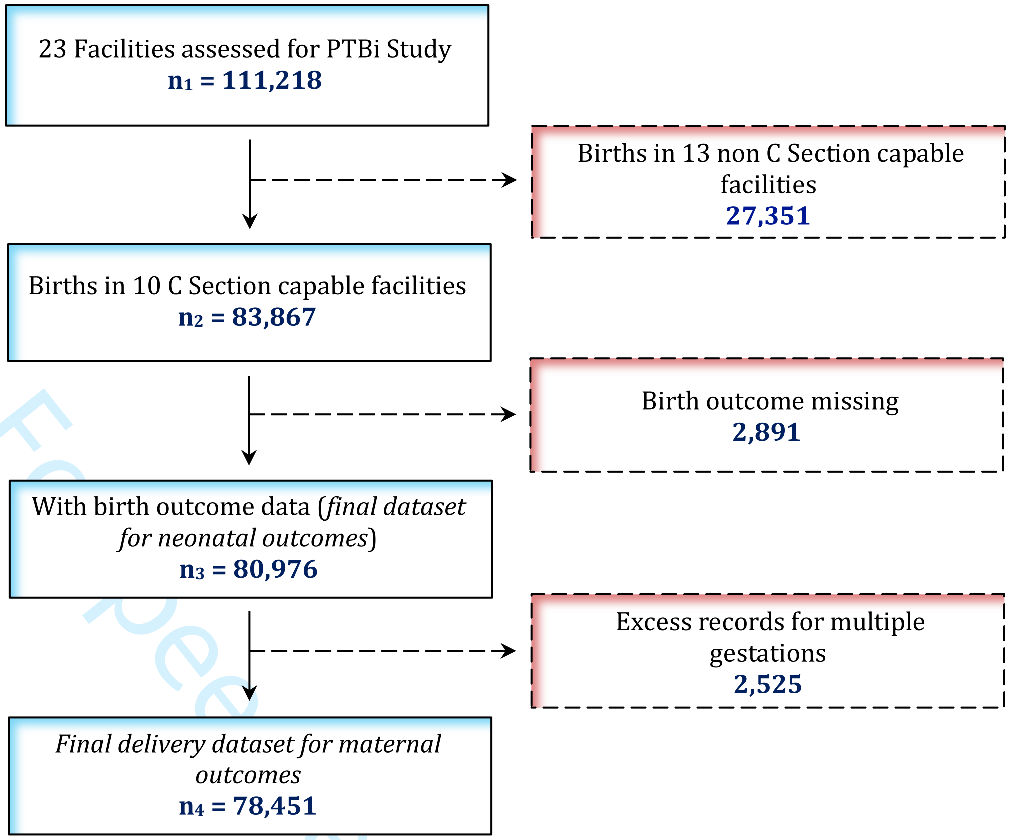

For peer review only

Figure 1: ***Adjusted odds ratios (aOR and 95% Confidence Interval) for facility-based pregnancy related death and cesarean section stratified by birth outcome and referral status.***[!]

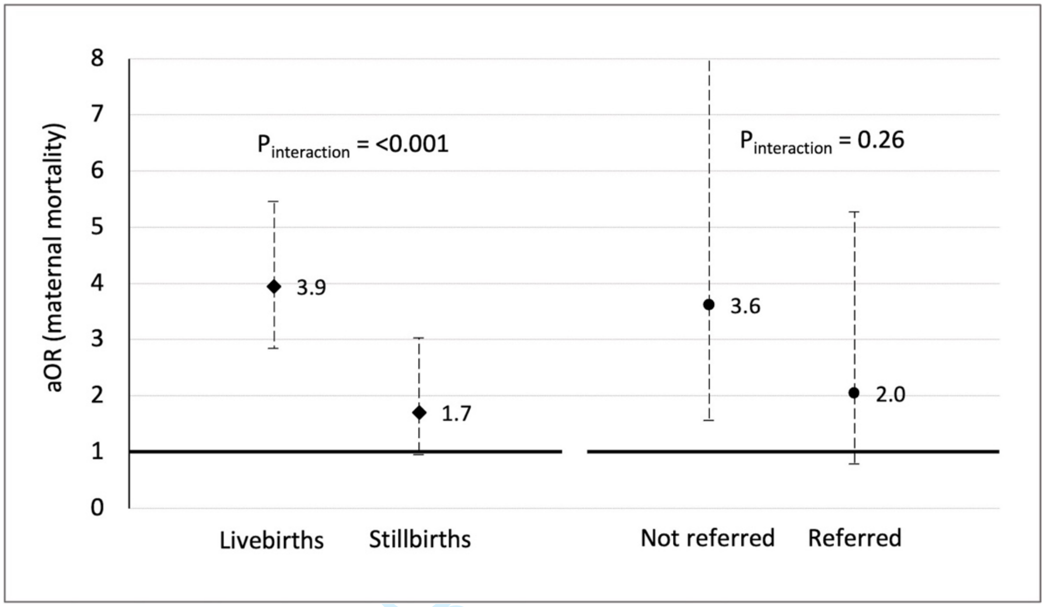

! Adjusted for birth weight, annual delivery volume, type of facility, country and exposure to PTBi intervention.

Note – After adjusting for multiple comparison using False Discovery Rate method, the interaction p-values changed to 0.0003 and 0.29, respectively.

For peer review only

Figure 3: Adjusted odds ratios (aOR and 95% Confidence Interval) for adverse neonatal outcomes and cesarean section stratified by referral status.[!]

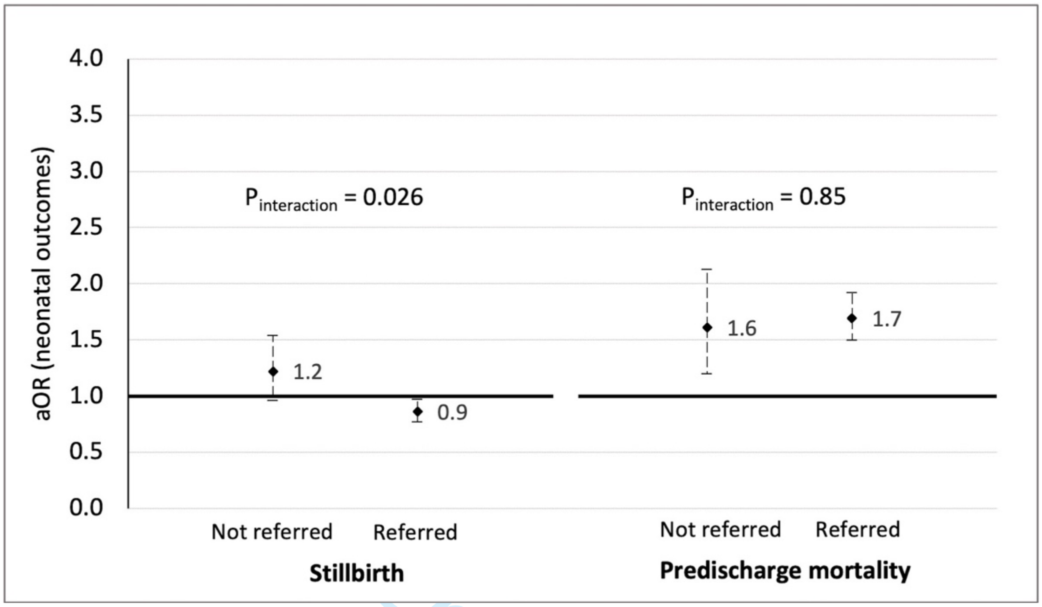

! Adjusted for birth weight, annual delivery volume, type of facility, country and exposure to PTBi intervention.
Note – After adjusting for multiple comparison using False Discovery Rate method, the interaction p-values changed to 0.04 for stillbirth and remained unchanged at 0.85 for predischarge mortality.

For peer review only

Online supplement

The hypothesized relationship is between cesarean section and maternal mortality as shown by the thick red arrow in the online supplementary Figure S1. To keep the conceptual framework simple, we are showing the Directed Acyclic Graph (DAG) for one outcome only. We considered each potential confounder and discussed all the backdoor pathways connecting the exposure (cesarean section) to the outcome (maternal mortality) through the confounders. In other words, once that confounder is adjusted, those pathways will no longer exist, potentially eliminating confounding, estimating only the *direct effect* between exposure and the outcome. We assume that there is no confounder misclassification i.e., after adjustment for the confounder in the model there is no residual confounding left from that confounder. Further, the majority of these confounders are categorical, and in accordance with their biological relationship with the exposure and the outcome. Hence, their functional form is assumed to be adequate to account for confounding.

1. Adjusting for *multiple gestation* would eliminate all the backdoor paths connecting mortality to cesarean section, through PTBi intervention. The backdoor paths from viability and birth weight/gestational age (GA) through multiple gestation are also removed. In the backdoor path MMR – multiple gestation – cesarean section – birth weight/GA, cesarean section is a collider. Adjustment for multiple gestation is necessary because multiple gestation pregnancy is a clinical indication for cesarean section. However, this could lead to over adjustment by inflating the standard error, as cesarean section is a collider in this pathway. We will still include multiple gestation in the preliminary model.
2. Adjusting for *birth weight/GA* would eliminate the direct path from birth weight/ GA to cesarean section and the backdoor paths through PTBi intervention, maternal history (e.g., age, prior cesarean section), viability, complications that leads to maternal deaths. The pathway from birth weight/GA – complications – maternal history leading on to maternal deaths through several other variables in the DAG are blocked paths because complication is a collider. Irrespective, birth weight adjustment removes the direct path from cesarean section – birth weight/GA – complications and will be included in the model.
3. Adjusting for *delivery volume*, will remove the backdoor from MMR – delivery volume – cesarean section. Further, it removes two additional directed paths from delivery volume to facility type and country and will be included in the model.
4. *PTBi intervention* requires adjustment because of the backdoor path from maternal death – PTBi – cesarean section. It will also remove the directed paths from PTBi intervention – facility readiness, PTBi intervention – APGAR score, facility type – PTBi intervention and country – PTBi intervention.
5. *Facility type* requires adjustment for the backdoor path maternal death – facility type – cesarean section. This adjustment will also remove the directed path from country – facility type.
6. The last confounder necessary to adjust is *country* for the backdoor path MMR – country – cesarean section.
7. *Maternal complications* will determine the need for a cesarean section. Independent of those cases that require a cesarean section, maternal complications such as PPH are primary reasons for maternal deaths. Complete maternal complication data is unavailable and hence it is a limitation. Statistically it leaves the potential for residual confounding.
8. A close look at this stage of the model will reveal that *APGAR score* does not require adjustment. All the backdoor paths from maternal death to cesarean section are through APGAR and stillbirth, making APGAR a collider and all those are blocked paths.
9. All other paths from viability, *weekend delivery and facility readiness* are blocked paths where cesarean section is the collider. Hence, conceptually there is no need for adjustment of any other confounder.
10. Of birth weight and GA, the final model had birth weight and not both because including GA leads to about loss of 8000 births, both are colinear (Pearson's correlation = 0.5) and the AICs for both models are close (AIC for the birth weight model is 1779 and AIC for the GA model is 1693).
11. Statistically adjusting for multiple gestation did not change the exposure – outcome association. The association with MMR presented in table 2 changed to 3.31 (2.21, 4.97) after including multiple gestation in the reported model. Hence, multiple gestation was not retained in the final model.

For peer review only - http://bmjopen.bmj.com/site/about/guidelines.xhtml

Figure S1. Conceptual diagram describing the hypothesized relationship (red arrow) between cesarean section and maternal mortality and the potential confounding due to other factors.

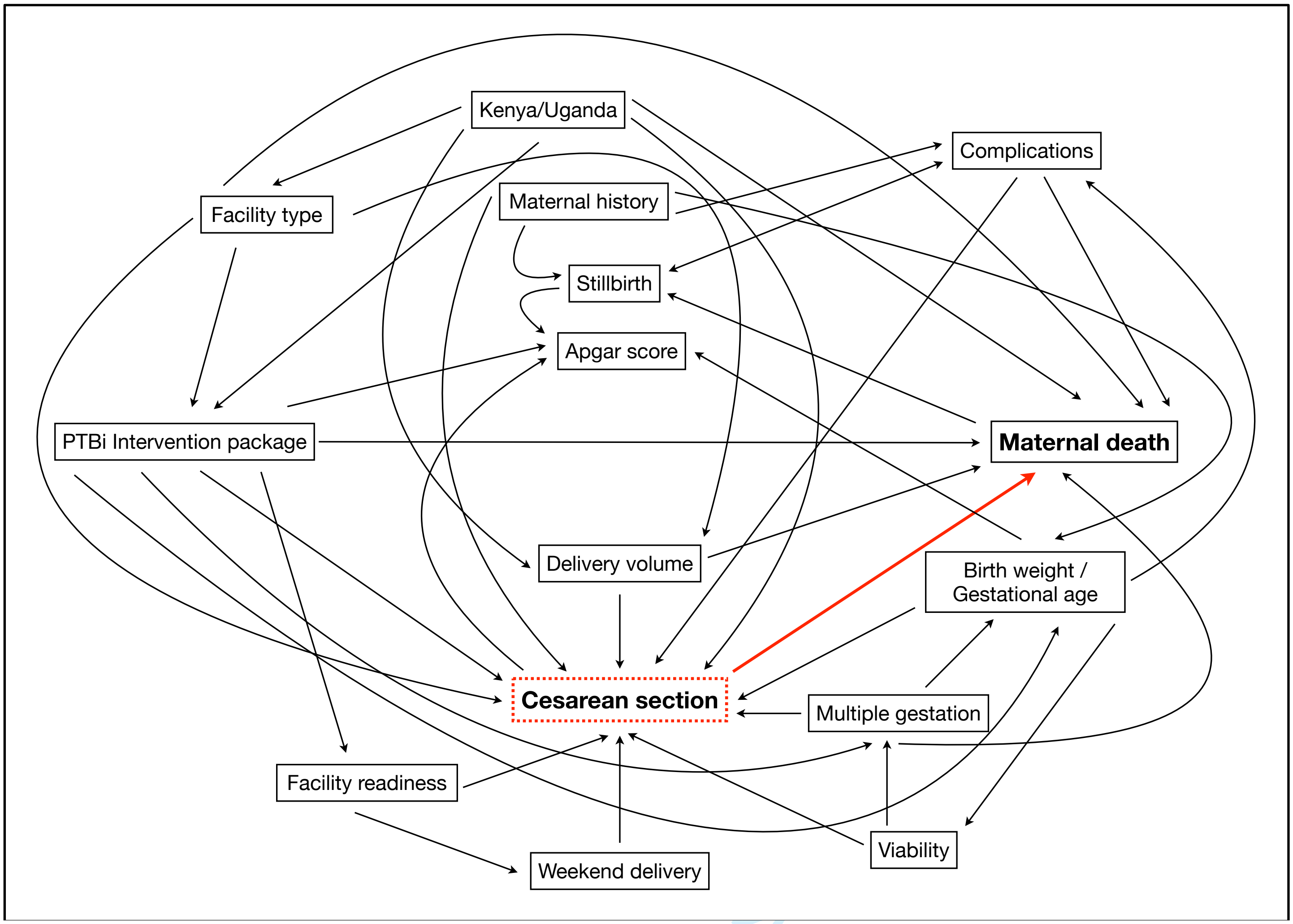

For peer review only - http://bmjopen.bmj.com/site/about/guidelines.xhtml

Table S1. Adjusted[!] odds ratios for types of stillbirths and cesarean sections.

	Vaginal Delivery % (n)	Cesarean Section % (n)	Adjusted Odds Ratio (95% CI)	P-value
**Antepartum/macerated stillbirth ***				
Live birth	75.7 (56,718)	24.3 (18,193)	1.00	
Yes	86.5 (276)	13.5 (43)	0.42 (0.30, 0.59)	<0.001
**Intrapartum/fresh stillbirth ***				
Live birth	75.7 (56,718)	24.3 (18,193)	1.00	
Yes	66.1 (232)	33.9 (119)	1.37 (1.10, 1.71)	0.005
**Undefined stillbirth ***				
Live birth	75.7 (56,718)	24.3 (18,193)	1.00	
Yes	67.5 (1,776)	32.5 (856)	1.37 (1.26, 1.49)	<0.001

! Adjusted for birth weight, annual delivery volume, type of facility, country and exposure to PTBi intervention. The models are single-level logistic regression.
** Based on the neonatal dataset of 80,976*

Table S2. Associations between pregnancy related maternal death and cesarean section stratified by birth outcome and referral status.[!]

		**Maternal mortality** (C Section × Stillbirths, $P_{interaction}$ <0.001*)		**Maternal mortality** (C Section × Referral Status, $P_{interactions}$ = 0.26 and 0.80*)		
		**Livebirths**	**All stillbirths**	**Not referred**	**Referred**	**Missing**
	n	66,098	2,954	39,519	6,482	21,472
**C Section** **	No	1.00	1.00	1.00	1.00	1.00
	Yes	3.94 (2.84, 5.46)	1.70 (0.95, 3.03)	3.61 (1.56, 8.37)	2.04 (0.79, 5.27)	3.10 (2.06, 4.65)
	FDR Unadjusted p-value	<0.001	0.07	0.003	0.14	0.001
	FDR adj p-value	0.0003	0.09	0.006	0.16	0.002
**Number of events**						
**C Section**	No	32	37	28	8	31
	Yes	44	25	33	12	22

! Adjusted for birth weight, annual delivery volume, type of facility and country.
** Interaction between the two variables and the p-value for the interaction term. For Referral Status, two p-values are reported because the variable has 3 categories. After adjusting for multiple comparison using False Discovery Rate (FDR) method, the FDR unadjusted interaction p-values changed from <0.001 to 0.0003 for stillbirth and from 0.26 to 0.29 and 0.80 to 0.83 for referral status.*

For peer review only - http://bmjopen.bmj.com/site/about/guidelines.xhtml

Table S3. Associations between adverse neonatal outcomes and cesarean section stratified by referral status.[!]

	**Stillbirth** (C Section × Referral Status, P interaction = 0.026 and 0.046*)			**Pre-discharge neonatal mortality** (C Section × Referral Status, P interaction = 0.85 and 0.003*)		
	**Not referred**	**Referred**	**Missing**	**Not referred**	**Referred**	**Missing**
n	42,813	7,007	27,625	43,349	7,110	27,625
**C Section**** No	1.00	1.00	1.00	1.00	1.00	1.00
Yes	1.22 (0.96, 1.54)	0.86 (0.77, 0.97)	1.74 (1.22, 2.46)	1.61 (1.37, 1.90)	1.69 (1.11, 2.59)	2.31 (1.82, 2.93)
FDR Unadj pval	0.10	0.012	0.002	<0.001	0.014	<0.001
FDR adj p-value	0.12	0.019	0.005	0.003	0.021	0.003
No. of events						
C Section. No	1,068	337	879	353	87	234
Yes	445	241	332	193	108	124

! Adjusted for birth weight, annual delivery volume, type of facility and country.

** Interaction between the two variables and the p-values for the interaction terms. Two p-values are reported because the referral variable has 3 categories. After adjusting for multiple comparison using False Discovery Rate (FDR) method, the interaction p-values changed to 0.04 and 0.06 for stillbirth, and 0.85 and 0.006 for predischarge mortality. These p-values should be compared to the ones in the very top row.*

Table S4. Association between Apgar score at 5 minutes and cesarean section stratified by referral status.[!]

	**Apgar <7** (C Section × Referral Status, P interaction <0.001 and 0.69*)		
	**Not referred**	**Referred**	**Missing**
n	35,075	6,218	22,651
**C Section**** No	1.00	1.00	1.00
Yes	1.96 (1.55, 2.48)	1.24 (1.05, 1.47)	2.02 (1.41, 2.89)
FDR Unadj pval	<0.001	0.01	<0.001
FDR adj p-value	0.0003	0.02	0.0003
No. of events			
C Section. No	682	293	974
Yes	471	303	413

! Adjusted for birth weight, annual delivery volume, type of facility and country.

** Interaction between the two variables and the p-values for the interaction terms. Two p-values are reported because the referral variable has 3 categories. After adjusting for multiple comparison using False Discovery Rate (FDR) method, the interaction p-values changed to 0.0003 and 0.74 for Apgar. These p-values should be compared to the ones in the very top row.*

For peer review only

Stillbirth, neonatal, and maternal mortality among cesarean births in Kenya and Uganda: a register-based prospective cohort study

Journal:	*BMJ Open*
Manuscript ID	bmjopen-2021-055904.R1
Article Type:	Original research
Date Submitted by the Author:	01-Mar-2022
Complete List of Authors:	Ghosh, Rakesh; University of California San Francisco, Institute for Global Health Sciences<br>Santos, Nicole; University of California San Francisco, Institute for Global Health Sciences<br>Butrick, Elizabeth ; University of California San Francisco<br>Wanyoro, Anthony; Kenyatta University, Department of OB/GYN<br>Waiswa, Peter; Makerere University, School of Public Health<br>Kim, Eliana; University of California San Francisco<br>Walker, Dilys; University of California San Francisco Department of Obstetrics Gynecology and Reproductive Sciences; University of California San Francisco Global Health Sciences
**Primary Subject Heading**:	Obstetrics and gynaecology
Secondary Subject Heading:	Epidemiology, Global health, Public health
Keywords:	Epidemiology < TROPICAL MEDICINE, OBSTETRICS, Quality in health care < HEALTH SERVICES ADMINISTRATION & MANAGEMENT

I, the Submitting Author has the right to grant and does grant on behalf of all authors of the Work (as defined in the below author licence), an exclusive licence and/or a non-exclusive licence for contributions from authors who are: i) UK Crown employees; ii) where BMJ has agreed a CC-BY licence shall apply, and/or iii) in accordance with the terms applicable for US Federal Government officers or employees acting as part of their official duties; on a worldwide, perpetual, irrevocable, royalty-free basis to BMJ Publishing Group Ltd ("BMJ") its licensees and where the relevant Journal is co-owned by BMJ to the co-owners of the Journal, to publish the Work in this journal and any other BMJ products and to exploit all rights, as set out in our licence.

The Submitting Author accepts and understands that any supply made under these terms is made by BMJ to the Submitting Author unless you are acting as an employee on behalf of your employer or a postgraduate student of an affiliated institution which is paying any applicable article publishing charge ("APC") for Open Access articles. Where the Submitting Author wishes to make the Work available on an Open Access basis (and intends to pay the relevant APC), the terms of reuse of such Open Access shall be governed by a Creative Commons licence – details of these licences and which Creative Commons licence will apply to this Work are set out in our licence referred to above.

Other than as permitted in any relevant BMJ Author's Self Archiving Policies, I confirm this Work has not been accepted for publication elsewhere, is not being considered for publication elsewhere and does not duplicate material already published. I confirm all authors consent to publication of this Work and authorise the granting of this licence.

For peer review only

Stillbirth, neonatal, and maternal mortality among cesarean births in Kenya and Uganda: a register-based prospective cohort study

Rakesh Ghosh[1*§], Nicole Santos[1*], Elizabeth Butrick[1], Anthony Wanyoro[2], Peter Waiswa[3,4], Eliana Kim[5], Dilys Walker[1,6]

1. University of California, San Francisco, Institute for Global Health Sciences, USA
2. Kenyatta University, Department of OB/GYN, Kenya
3. Makerere University, School of Public Health, Uganda
4. Department of Global Public Health, Karolinska Institutet, Sweden
5. University of California, San Francisco, School of Medicine, USA
6. University of California, San Francisco, Department of OB/GYN and Reproductive Sciences, USA

* These authors contributed equally to this work.
§ Corresponding author: Rakesh Ghosh, Institute for Global Health Sciences, University of California San Francisco, 550 16th St, San Francisco, CA, 94158, USA. Email: Rakesh.Ghosh@ucsf.edu

Word count – 3667 (main text only not including references)

For peer review only

ABSTRACT (300 words max)

Objective: To investigate the interaction of risks for adverse maternal and perinatal outcomes (stillbirth, pre-discharge neonatal and maternal mortality) among cesarean section (CS) compared to vaginal deliveries (VD).

Design: Prospective cohort study.

Setting: 10 CS-capable facilities in Busoga Region, East-Central Uganda and Migori County, Kenya.

Participants: Individual birth data were extracted from maternity registers between October 2016 and April 2019. There were a total of 77,242 livebirths and 3,734 stillbirths. Overall, 24% of deliveries were by CS with a range of 9% to 49% across facilities.

Primary outcome measures: Stillbirth, pre-discharge neonatal mortality and maternal mortality.

Results: The adjusted odds ratios for stillbirth, pre-discharge neonatal mortality and maternal mortality after a CS were 1.3 (95% CI: 1.1, 1.6), 1.9 (95% CI: 1.6, 2.2) and 3.3 (95% CI: 2.2, 4.9), respectively, compared to a VD. The association between maternal mortality and CS was 3.9 (95% CI: 2.8, 5.5) when the delivery was a livebirth and 1.7 (95% CI: 1.0, 3.0) when it was a stillbirth. Post-hoc analyses showed that mothers who received a CS had a lower risk of stillbirth if they were documented as a referral.

Conclusion: In this context, CS births were at higher risk for worse outcomes compared to VD. Better understanding of CS use and associated adverse outcomes within the mother-baby dyad is necessary to identify opportunities to improve quality of intrapartum care.

Clinical trial registration: This is a secondary analysis of data from a trial registered with Clinical Trials.gov, NCT03112018

For peer review only

For peer review only - http://bmjopen.bmj.com/site/about/guidelines.xhtml

Strengths and limitations of the study

- This study uses data from over 80,000 births across 10 facilities in Kenya and Uganda with a range of cesarean section rates, to compare risk of intrapartum stillbirths, pre-discharge neonatal mortality, and facility-based pregnancy-related maternal death among women who underwent cesarean section compared to women who delivered vaginally.
- This study explores risk of adverse outcomes among women who were referred in, and uncovers potential avenues of future exploration.
- We lacked data on various maternal characteristics such as Robson criteria for cesarean section, antenatal care coverage, indication for cesarean section, peri- and post-operative complications, and cause of death.
- We also lacked data on system-related factors such as time between cesarean section decision and incision, access to anesthesia, and personnel conducting the cesarean section.

Funding statement: This work was supported by the East Africa Preterm Birth Initiative, which was funded by the Bill and Melinda Gates Foundation (OPP1107312). Under the grant conditions of the Foundation, a Creative Commons Attribution 4.0 Generic License has already been assigned to the Author Accepted Manuscript version that might arise from this submission.

Keywords: cesarean section; maternal mortality, neonatal mortality; stillbirth

Competing interests: none declared

For peer review only

INTRODUCTION

As facility-based births increase, access to and performance of cesarean section (CS) is also increasing in many low- and middle-income countries (LMICs). However, while global CS rates increased from 12% in 2000 to 21% in 2015, the increase has been only 5% to 6% in East and South Africa.[1] CS rates are projected to near 30% globally by 2030, though Sub-Saharan Africa is estimated to remain below 8%.[2]

Low CS rates may indicate unmet need and missed opportunity to prevent adverse outcomes.[3,4] Evidence from 16 Sub-Saharan African countries between 2008 and 2011 showed that an average CS rate of approximately 4% was associated with increased maternal, neonatal and infant mortality, which may have been averted by increasing access to cesarean delivery for medically indicated cases.[5] Additionally, it is well-documented that lack of trained personnel or access to safe anesthesia, blood supply and essential drugs, contribute to CS-related adverse outcomes in many LMICs.[5–7] A review of 196 studies from 67 LMICs showed that rates of maternal death and stillbirth were 8 and 57 per 1000 CS procedures, and were highest in sub-Saharan Africa, at 11 and 83, respectively.[8] Given that CS is usually indicated for a maternal or neonatal complication, it is not surprising that CS is associated with higher maternal and neonatal mortality. Access to timely and safe CS is critical to avert poor outcomes related to the mother-baby dyad.

Increasing CS rates to recommended levels, especially in resource limited settings, may not always confer incremental benefit.[9–11] Overuse of CS in such settings introduce unnecessary risk for both mothers and newborns by diverting facility resources from the clinically indicated cases to low-risk pregnancies.[6,12]

While evidence separately links CS with maternal mortality and CS with neonatal outcomes in different settings, no data, to our knowledge, describes the inter-relationship between maternal mortality and stillbirth among births by CS. Generally, if intrauterine demise is identified prior to delivery, there are few indications to perform a CS due to increased maternal risks.[13] To better understand this interaction, we investigated the

For peer review only

characteristics of CS performed across 10 facilities in Kenya and Uganda in a prospective birth cohort. In Uganda, facility-based CS rates were 22–32% in general hospitals and 20–25% in referral hospitals between 2012 and 2016.[14] In Kenya, 2014 Demographic and Health Survey (DHS) data showed an overall 13% CS rate among institutional births, with notable disparities between rural and urban populations and across socioeconomic levels.[15] Data from the East Africa Preterm Birth Initiative (PTBi), a cluster-randomized trial (CRCT), provides a unique opportunity to investigate maternal and neonatal outcomes in the CS capable facilities in the two countries.[16,17] In this study we investigated the interaction of risks for adverse maternal and perinatal outcomes (stillbirth, pre-discharge neonatal and maternal mortality) among CS compared to vaginal deliveries (VD).

METHODS

Study design and setting

This analysis used maternity register data for deliveries between October 2016 and April 2019 from 10 CS-capable facilities in Busoga Region, East-Central Uganda and Migori County, Kenya that were involved in a pair-matched CRCT conducted by the East Africa Preterm Birth Initiative (trial registration: NCT03112018).[16,17] Four facilities were private not-for-profit mission hospitals and six were public hospitals with 620-2420 and 1200-6700 deliveries per year, respectively. Four of the 10 facilities that are part of this study were in the control arm and three were in the intervention arm of the CRCT. The three remaining hospitals were large referral hospitals that received the intervention but were not part of the CRCT given the inability to pair match them.

Busoga Region and Migori County have a predominantly rural population of approximately three million and one million, respectively. Most women in the two respective regions deliver in a facility (77% and 53%), though regional neonatal mortality rates (27 and 19 per 1000 live births) and stillbirth rates (17 and 10 per 1000 pregnancies) remain high.[18,19] In Uganda, health centers (level III) offer basic emergency obstetric care and are

For peer review only - http://bmjopen.bmj.com/site/about/guidelines.xhtml

linked to a referring facility (either a health center IV or a hospital) for comprehensive emergency obstetric care. In Kenya, referral to the county referral hospital from lower-level facilities is standard, though women may seek services within the private sector or across county borders.

Data sources, eligibility criteria and variables of interest

Anonymized individual level birth data were extracted monthly from facility maternity registers by PTBi study staff. These routine data sources are completed by frontline health care workers. As part of the CRCT, all facilities received data strengthening, including reinforcement of key indicator definitions and continuous feedback on maternity register completion and accuracy for key variables (e.g. gestational age, birth weight, Apgar, discharge status).[20,21] There was no routine follow-up after discharge.

Data collected include maternal (age, multiplicity, referral status) and newborn (birth weight, gestational age at birth, five-minute Apgar, infant sex) characteristics and maternal and newborn status at discharge. Maternal length of hospital stay was calculated using delivery and discharge dates. Mode of delivery was documented as CS, normal vaginal, assisted vaginal, breech, and vacuum extraction. For this analysis, births were excluded if mode of delivery was missing, or documented as an abortion or vacuum aspiration. Babies born before arrival to the facility were also excluded.

The main outcomes were defined as: (1) stillbirths (birth at ≥28 weeks of gestation with no signs of life); (2) pre-discharge neonatal mortality (death of a liveborn baby before facility discharge); and (3) facility-based pregnancy-related maternal death.

Birth outcome was determined by triangulating data from delivery status, baby discharge status, and Apgar scores at one and five minutes. In both countries, stillbirths were categorized into intrapartum stillbirth (intrauterine death of a fetus during labour or delivery; recorded as “fresh stillbirth”) or antepartum stillbirth (intrauterine death of a fetus before the onset of labour, where the fetus showed degenerative changes,

For peer review only - http://bmjopen.bmj.com/site/about/guidelines.xhtml

recorded as "macerated stillbirth").[22] If either the delivery status or the baby discharge status were recorded as antepartum stillbirth or intrapartum stillbirth and the Apgar scores at one *and* five minutes were zero, their birth outcome was designated as documented. To reconcile the heterogeneity all stillbirths were categorized into a single group.

Statistical analysis

The association of CS with maternal and perinatal outcomes was quantified using logistic regression and results are presented as odds ratios (OR) with 95% confidence intervals (95% CI). The data were two-tiered – individual deliveries clustered within facilities. We used mixed effect models with a random intercept for each facility to account for the non-independence of the deliveries within facilities. When stratified by types of stillbirth, the number of outcomes reduced, and the models did not converge. Hence, we fit single-level logistic regression to gain insight into how the associations differ by stillbirth sub-types (supplemental results only).

To specify a parsimonious model, we used a directed acyclic graph (DAG)[23] and change-in-estimate confounder selection method.[24] The DAG identified a necessary and sufficient set of potential confounders that required adjustment. We then used the change-in-estimate method of statistical modelling to identify the final set of potential confounders, with 5% change in the exposure-outcome association as the criterion. More explanation about the statistical modelling and the directed paths are presented in Figure S1.

Interaction was investigated using a product term in the models. If the product term was significant at the 5% significance level, the models were stratified by the interacting variable. Led by the descriptive results on referrals, we framed a post-hoc objective to examine interaction between CS and referral status on adverse outcomes. To minimize chances of false-positive associations, we adjusted for multiple comparisons using false discovery rate.[25] The analysis was conducted in STATA 16.1.

Ethical considerations

For peer review only - http://bmjopen.bmj.com/site/about/guidelines.xhtml

The PTBi EA CRCT was granted ethical approvals from Higher Degrees, Research and Ethics Committee (MUSPH HDREC 395) from Makerere University; KEMRI Scientific and Ethics Review Committee (KEMRI/SERU/CCR/0034/3251) and UCSF Committee on Human Research (16-19162). Permission to extract non-identifiable data from maternity registers was allowed under these approvals.

Patient and public involvement

Our research question was informed by an observation that intrapartum stillbirth rates were high, especially among women who had CS. Since undergoing a CS involves added risk and a longer recovery period, we expect that women are more likely to consent to a CS if they anticipate a greater chance of an improved outcome. Given CS is often done for complications that lead to poorer outcomes, we decided to assess CS and maternal and perinatal outcomes in this context. We abstracted delivery and newborn care data for all women who delivered during the study period from the facility maternity register, under an IRB-approved waiver of consent. As this was a secondary analysis of a CRCT, all women who gave birth at a given facility, received the same standard of care. Patients were not involved in the design or conduct of the study, other than being a participant. Study results were shared with participating facilities' administration and staff by our in-country teams. However, results will not be disseminated directly to women.

RESULTS

The final analytical dataset is shown in Figure 1. PTBi EA collected information on 83,867 deliveries across the 10 CS capable facilities, of which 80,976 had birth outcomes. To avoid double counting for multiple gestation pregnancies, we considered multiples as a single delivery event (n=78,451) for maternal outcomes.

For peer review only - http://bmjopen.bmj.com/site/about/guidelines.xhtml

There were 164 maternal deaths, 3,734 stillbirths and 77,242 live births. The maternal mortality ratio was 212 per 100,000 livebirths; the stillbirth rate was 46 per 1,000 births; and the pre-discharge neonatal mortality rate was 16 per 1,000 livebirths.

Overall, 24% of deliveries were by CS (19,155/78,451) with a range of 9% to 49% across facilities. Table 1 shows that private not-for-profit mission facilities performed more CS (38%) than public facilities (21%). Neither exposure to the PTBi intervention nor annual delivery volume were significantly associated with CS rates.

Maternal and neonatal characteristics by mode of delivery are shown in Table 1. There was an increased association between CS and referred-in status, compared to those not referred. The length of hospital stay after delivery was significantly associated with mode of delivery with about two-thirds of mothers who were hospitalized for seven days or longer having undergone a CS (OR 1.9, 95% CI: 1.8, 2.0). Overall, about 6% of women who underwent a CS had a hospital stay of more than seven days compared to <1% for vaginal delivery. The median (interquartile range) length of stay was 3 (1, 5) days for a CS, compared to 1 (0, 1) day for a VD. Over a third of the women (36%) who had multiple gestations underwent CS, with an OR of 1.8 (95% CI: 1.5, 2.0), relative to singletons.

For neonatal characteristics, relative to infants with gestational age (GA) between 37 and 40 weeks, those who were <32 completed weeks at birth had reduced odds for CS, while those between 32 and <37 weeks and >40 weeks had increased odds for CS (Table 1). Likewise, relative to infants between 2500 and 3999 grams, those weighing <1000 grams had reduced odds of CS, while those between 1000 and 2499 grams and those ≥4000 grams had increased odds of CS. There was an increased association between CS and Apgar <7 at five minutes (OR 1.8, 95% CI: 1.4, 2.5), compared to those with an Apgar score ≥7.

Table 2 presents the adjusted OR (aOR) for the association between CS and adverse maternal or perinatal health outcomes. The aORs were 1.3 (95% CI: 1.1, 1.6) for stillbirth and 1.9 (95% CI: 1.6, 2.2) for pre-discharge

For peer review only

neonatal mortality when the mode of delivery was CS compared to VD. The aOR for maternal death after a CS was 3.3 times higher (95% CI: 2.2, 4.9) compared to VD. Table S1 presents CS associations by stillbirth types, which is decreased for antepartum stillbirth (0.7, 95% CI: 0.7, 0.8) and increased for intrapartum stillbirth (1.8, 95% CI: 1.6, 2.0).

Figure 2 shows that the risk for maternal death after CS was higher for a livebirth than for a stillbirth. For livebirths, the aOR for maternal death after CS was 3.9 (95% CI: 2.8, 5.5), compared to VD, while it was 1.7 (95% CI: 1.0, 3.0) for stillbirths. For maternal mortality after CS, the association did not statistically differ by referral status of the mothers. Numerical estimates are presented in Table S2.

Figure 3 shows that when the mother was not referred, the aOR for stillbirth at CS was 1.2 (95% CI: 1.0, 1.5), compared to a VD and 0.9 (95% CI: 0.8, 1.0) when the delivery was referred-in. For pre-discharge neonatal mortality, the association did not statistically differ by referral status of the mothers. Table S3 presents numerical estimates for Figure 3.

Apgar score <7 at five minutes is an indicator of neonatal well-being, as well as success of resuscitation. Table S3 shows that when the mother was not referred, the odds for Apgar <7 after CS was 2.0 (95% CI: 1.6, 2.5), compared to a VD and 1.2 (95% CI: 1.1, 1.5) when the delivery was referred-in.

The statistical significance for the associations presented in Table 2 as well as the interactions remained largely unchanged after adjustment for multiple comparisons (Tables S2 and S3).

DISCUSSION

This analysis demonstrates an increased association of CS with adverse maternal and perinatal outcomes in two sub-Saharan countries. Compared to women who had a VD, those who underwent CS had increased association with maternal death, stillbirth and pre-discharge neonatal mortality. We found a higher association

For peer review only - http://bmjopen.bmj.com/site/about/guidelines.xhtml

between CS and maternal mortality for a livebirth than for a stillbirth. This novel differential association by birth outcome is subject to independent verification but underscores the need to approach the clinical situation and the decision-making from the lens of the mother-baby dyad, that optimizes improved outcomes. We also found that women who were documented as referred-in and underwent a CS had a lower risk of stillbirth than those who were not referred-in and underwent a CS. While the risk for maternal mortality after a CS was not statistically different by referral status, it generally trended towards lower risk of poor outcome if documented as referred-in.

Our overall finding that women who had a CS had higher risk of maternal death is consistent with previous studies and not surprising given the indications for CS include high-risk conditions for both mothers and newborns. In a South African study, CS rates at various facilities ranged between 15% and 28% and the risk of maternal death after CS was 2.8 times that of VD.[26] A study across 41 referral hospitals in Mali and Senegal demonstrated that women who underwent intrapartum CS were at higher risk for maternal mortality compared to women who had a spontaneous VD, as well as pre-discharge neonatal mortality.[27]

Our findings suggest the risk of maternal death after a CS was higher for a livebirth than it was for a stillbirth. With limited relevant data in the registries and no access to maternal death reviews, we could not ascertain the cause and time of death, associated complications, and time between decision to incision, limiting the ability to interpret these results. At Mulago National Referral Hospital in Uganda, shorter decision-to-CS delivery times were associated with fetal distress, while longer times were associated with pre-eclampsia and premature rupture of membranes (PROM), hinting towards increased attention and action to ensure fetal well-being.[28] This could be because mothers who have pre-eclampsia or PROM may require stabilization or antenatal corticosteroids prior to CS, unlike mothers who are diagnosed with non-reassuring fetal heart tones. However, elevated odds of maternal death even with stillbirth (but less than livebirth) bring to light the need for careful evaluation on indications for cesarean and quality of cesarean care.

For peer review only - http://bmjopen.bmj.com/site/about/guidelines.xhtml

We found higher odds of stillbirth among women who received a CS compared to VD. Studies have linked unmet need for CS to increased intrapartum stillbirths.[4,29] In this study, more than 30% of stillbirths were delivered by CS suggesting that intrapartum quality of care and CS timeliness may contribute to stillbirth outcomes. Nonetheless, it raises questions of adequate fetal monitoring prior to CS initiation, appropriate indication for CS, and timeliness. It is unclear whether these were conducted for clinically appropriate indications, such as uterine rupture, prior CS, or if there was a lack of alternative capacity to manage stillbirth. This paucity of data highlights the value of auditing CS cases particularly those in which a stillbirth is delivered. This may reveal important and urgent opportunities for quality improvement of intrapartum care in these settings.

Increased CS rates among referred-in mothers could contribute to higher rates of stillbirths[30,31] and maternal deaths,[26] due to delays in transportation, timeliness, etc. However, we were intrigued to note that referred-in mothers had a lower risk of stillbirth and maternal death, though the latter was not significant. We also found that compromised newborn well-being at birth (Apgar score <7 at five minutes) showed a similar relationship, suggesting that referred-in deliveries may have been exposed to more timely intervention or increased evidence-based practices during the intrapartum period, such as newborn resuscitation. While the underlying contributors to this finding are unclear; some hypotheses might be: (1) referred-in women were stabilized prior to arrival; (2) prior assessment led to more timely CS upon arrival to the receiving facility; (3) referred-in women had more time to mobilize resources for a timely CS; and (4) decision to refer was made before the onset of severe complications (e.g. early diagnoses of CS indication) or due to non-medical reasons. Missingness of the referral variable demands cautious interpretation. However, the intriguing results suggest that the existing referral system may be functional in identifying and managing high-risk women and their newborns. At the least, this finding deserves further exploration in this particular referral system and in similar contexts for a deeper understanding.

For peer review only

For peer review only - http://bmjopen.bmj.com/site/about/guidelines.xhtml

Collectively, these findings highlight the need to assess gaps in CS quality of care and system bottlenecks to improve maternal and perinatal outcomes. CS-specific checklists and audits have improved uptake of evidence-based practices, clinical documentation and quality of care.[32,33] Few vacuum or forceps deliveries were recorded in maternity registers, which should be optimized to avoid unnecessary CS, particularly among known intrauterine fetal deaths.[34,35] Another obvious area of improvement is documentation of clinical diagnoses, indications for CS, and referral status. In the absence of robust data for clinical indications for CS, we cannot rule out elective CS, though clinically indicated CS likely constitute a vast majority of all CS in this context.[36] While PTBi EA invested in data strengthening across the study facilities, this largely focused on variables such as gestational age, birth weight, Apgar and discharge status.[16] Maternity registers use free text to document final diagnosis, delivery complications and referral status. In this study, among women who had CS and stillbirth, 40% had no diagnosis documented; 63% and 43% of women who died after a VD or a CS had no diagnosis, respectively. Due to these data issues, we cannot assess whether conditions such as pre- or peri-operative obstetric hemorrhage, hypertensive disorders, infection or anesthesia complications may have contributed to deaths, all of which have been independently associated with maternal mortality.[37] Given the relatively high CS rates in some of the facilities and the rising rates globally, inclusion of CS indications may be warranted in birth registries.

Likewise, differentiation between intrapartum and antepartum stillbirths would undoubtedly inform opportunities and curb the potential overuse of CS for non-indications, particularly in cases of known stillbirth. Overall, rate of intrapartum stillbirths has previously been suggested as a maternal quality care indicator by WHO and other stakeholders.[38] Disaggregating this indicator by mode of delivery could serve as a quality of intrapartum care indicator, particularly in the context of rising CS rates across LMICs[39] and the fact that CS should be considered a last resort for delivering a stillborn infant due to the increased risk to the mother.[13]

Strengths and Limitations

For peer review only - http://bmjopen.bmj.com/site/about/guidelines.xhtml

The study has several limitations. Outcomes were ascertained by facility personnel and there may be data quality issues as maternity registers are often completed in the context of high workload. For example, approximately 40 cases had conflicting delivery and discharge status, and were excluded. Second, we lacked robust data on various maternal characteristics such as Robson criteria for CS, antenatal care coverage, indication for CS, peri- and post-operative complications, and cause of death. We also lacked data on system-related factors such as time between CS decision and incision, access to anesthesia, and personnel conducting the CS. As noted above, this information would have helped contextualize our findings. CS and VD are very different by its very nature leaving little room for misclassification. Grouping all stillbirths into one, further reduces possibility of misclassification by type. However, there is room for potential misclassification between stillbirths and predischarge neonatal mortality, but such misclassification is unlikely to happen among the CS, as these are conducted by specialists.

Regardless of these limitations, we used robust design and analytic approaches to quantify associations and have been cautious in drawing conclusions. By leveraging existing data systems, we captured real-world contexts and underscore the importance of data standardization, completeness and accuracy. Another strength of this study was the availability of pregnancy-related mortality data across heterogeneous types of facilities in two countries. Given the duration of the PTBi EA program, we had sufficient sample size to power the investigation for maternal mortality and investigate a novel interaction between CS and birth outcome. This study provides rare evidence related to outcomes of CS from a low resource setting and the results may be generalizable to similar contexts and settings. The results are unlikely to be chance findings, given the significance level after adjustment for multiple comparisons.

CONCLUSION

For peer review only

For peer review only - http://bmjopen.bmj.com/site/about/guidelines.xhtml

In this study, CS births were at higher risk for worse maternal and perinatal outcomes compared to VD. Additionally, the risk of maternal death after a CS was higher among livebirths than it was for stillbirths. Better understanding of CS use and associated adverse outcomes within the mother-baby dyad is necessary to identify opportunities to improve quality of intrapartum care. Detailed case documentation including indications for CS, referral and causes of death is needed to shed light on these issues and identify pathways to improve care.

For peer review only

DECLARATIONS

Acknowledgements

We appreciate all the facility healthcare workers, leadership and administration who made this work possible. We thank all members of the Preterm Birth Initiative Kenya & Uganda Implementation Research Collaborative, particularly the data teams who helped strengthen and collect data from facility maternity registers. We thank Phelgona Otieno who was the Kenya PI for the parent trial. In particular, we convey our heartfelt thanks to the study participants for their participation and immensely valuable contribution to this study.

Author contributions

DW, EB, NS and RG contributed to study conceptualization. RG and EK helped in data cleaning and preparing the data for analysis. RG led data analysis. RG and NS drafted and revised the manuscript. DW, PW, AW, EK, EB, NS and RG contributed to data synthesis and interpretation, review and approval of the final version of the manuscript for submission. DW, PW as PIs for the parent trial were responsible for overall design, implementation and data collection.

Details of Ethics Approval

The East Africa Preterm Birth Initiative was granted ethical approvals from Higher Degrees, Research and Ethics Committee (MUSPH HDREC 395) from Makerere University; KEMRI Scientific and Ethics Review Committee (KEMRI/SERU/CCR/0034/3251) and UCSF Committee on Human Research (16-19162). Permission to extract non-identifiable data from maternity registers was allowed under these approvals.

Funding

This work was supported by the East Africa Preterm Birth Initiative, which was funded by the Bill and Melinda Gates Foundation (OPP1107312). The funders did not have input on analysis or interpretation of results.

Disclosure of interests

All authors declare no conflict of interest.

Data sharing statement

Anonymized data will be made available upon reasonable request to: Dilys.Walker@ucsf.edu

ew only

For peer review only - http://bmjopen.bmj.com/site/about/guidelines.xhtml

REFERENCES

1. Boerma T, Ronsmans C, Melesse DY, Barros AJD, Barros FC, Juan L, et al. Global epidemiology of use of and disparities in caesarean sections. Lancet [Internet]. 2018;392(10155):1341–8. Available from: http://dx.doi.org/10.1016/S0140-6736(18)31928-7

2. Betran AP, Ye J, Moller B, Souza JP. Trends and projections of caesarean section rates : global and regional estimates. 2021;1–8.

3. Belizán JM, Minckas N, McClure EM, Saleem S, Moore JL, Goudar SS, et al. An approach to identify a minimum and rational proportion of caesarean sections in resource-poor settings: a global network study. Lancet Glob Heal. 2018;6(8):e894–901.

4. Harrison MS, Pasha O, Saleem S, Ali S, Chomba E, Carlo WA, et al. A prospective study of maternal, fetal, and neonatal outcomes in the setting of cesarean section in low- and middle-income countries. Acta Obs Gynecol Scand. 2018;96(4):410–20.

5. Irani M, Deering S. Challenges affecting access to cesarean delivery and strategies to overcome them in low-income countries. Int J Gynecol Obstet. 2015;131(1):30–4.

6. Harrison MS, Goldenberg RL. Cesarean section in sub-Saharan Africa. Matern Heal Neonatol Perinatol [Internet]. 2016;2(1):1–10. Available from: http://dx.doi.org/10.1186/s40748-016-0033-x

7. Epiu I, Wabule A, Kambugu A, Mayanja-Kizza H, Tindimwebwa JVB, Dubowitz G. Key bottlenecks to the provision of safe obstetric anaesthesia in low- income countries; a cross-sectional survey of 64 hospitals in Uganda. BMC Pregnancy Childbirth. 2017;17(1):1–8.

8. Sobhy S, Arroyo-Manzano D, Murugesu N, Karthikeyan G, Kumar V, Kaur I, et al. Maternal and perinatal mortality and complications associated with caesarean section in low-income and middle-income countries: a systematic review and meta-analysis. Lancet [Internet]. 2019;393(10184):1973–82. Available from: http://dx.doi.org/10.1016/S0140-6736(18)32386-9

9. Betrán AP, Temmerman M, Kingdon C, Mohiddin A, Opiyo N, Torloni MR, et al. Interventions to reduce unnecessary caesarean sections in healthy women and babies. Lancet [Internet]. 2018;392(10155):1358–68. Available from: http://dx.doi.org/10.1016/S0140-6736(18)31927-5

10. WHO. WHO Statement on Caesarean Section Rates [Internet]. 2015. Available from: https://www.who.int/reproductivehealth/publications/maternal_perinatal_health/cs-statement/en/

11. Ye J, Zhang J, Mikolajczyk R, Torloni MR, Gülmezoglu AM, Betran AP. Association between rates of caesarean section and maternal and neonatal mortality in the 21st century: A worldwide population-based ecological study with longitudinal data. BJOG An Int J Obstet Gynaecol. 2016;123(5):745–53.

12. Litorp H, Kidanto HL, Nystrom L, Darj E, Essén B. Increasing caesarean section rates among low-risk groups: A panel study classifying deliveries according to Robson at a university hospital in Tanzania. BMC Pregnancy Childbirth. 2013;13:1–10.

13. Chakhtoura NA, Reddy UM. Management of stillbirth delivery. Semin Perinatol [Internet]. 2015;39(6):501–4. Available from: http://dx.doi.org/10.1053/j.semperi.2015.07.016

14. Atuheire EB, Opio DN, Kadobera D, Ario AR, Matovu JKB, Harris J, et al. Spatial and temporal trends of cesarean deliveries in Uganda: 2012-2016. BMC Pregnancy Childbirth. 2019;19(1):1–8.

15. Ochieng Arunda M, Agardh A, Asamoah BO. Cesarean delivery and associated socioeconomic factors and

For peer review only - http://bmjopen.bmj.com/site/about/guidelines.xhtml

neonatal survival outcome in Kenya and Tanzania: analysis of national survey data. Glob Health Action [Internet]. 2020;13(1). Available from: https://doi.org/10.1080/16549716.2020.1748403

16. Walker D, Otieno P, Butrick E, Namazzi G, Achola K, Merai R, et al. Effect of a quality improvement package for intrapartum and immediate newborn care on fresh stillbirth and neonatal mortality among preterm and low-birthweight babies in Kenya and Uganda: a cluster-randomised facility-based trial. Lancet Glob Heal [Internet]. 2020;8(8):e1061–70. Available from: http://dx.doi.org/10.1016/S2214-109X(20)30232-1

17. Otieno P, Waiswa P, Butrick E, Namazzi G, Achola K, Santos N, et al. Strengthening intrapartum and immediate newborn care to reduce morbidity and mortality of preterm infants born in health facilities in Migori County, Kenya and Busoga Region, Uganda: A study protocol for a randomized controlled trial. Trials. 2018;19(1).

18. Kenya Demographic and Health Survey 2015. Nairobi, Kenya and Rockville, Maryland, USA; 2015.

19. Uganda Demographic and Health Survey 2016. Kampala, Uganda and Rockville, Maryland, USA; 2018.

20. Keating R, Merai R, Mubiri P, Kajjo D, Otare C, Mugume D, et al. Assessing effects of a data quality strengthening campaign on completeness of key fields in facility-based maternity registers in Kenya and Uganda. East Africa J Appl Heal Monit Eval. 2019;1–7.

21. Waiswa P, Higgins B V., Mubiri P, Kirumbi L, Butrick E, Merai R, et al. Pregnancy outcomes in facility deliveries in Kenya and Uganda: A large cross-sectional analysis of maternity registers illuminating opportunities for mortality prevention. PLoS One [Internet]. 2020;15(6):1–17. Available from: http://dx.doi.org/10.1371/journal.pone.0233845

22. Lawn JE, Blencowe H, Waiswa P, Amouzou A, Mathers C, Hogan D, et al. Stillbirths: rates, risk factors, and acceleration towards 2030. Lancet. 2016 Feb;387(10018):587–603.

23. Greenland S, Pearl J, Robins JM. Causal diagrams for epidemiologic research. Vol. 10, Epidemiology. 1999. p. 37–48.

24. Analysis E. Commentary Modeling and Variable Selection in Epidemiologic Analysis SANDER GREENLAND, MS, DrPH. 1989;79(3):1–21.

25. Storey JD. The positive false discovery rate: A Bayesian interpretation and the q-value. Ann Stat. 2003;31(6):2013–35.

26. Gebhardt GS, Fawcus S, Moodley J, Farina Z. Maternal death and caesarean section in South Africa: Results from the 2011 - 2013 saving mothers report of the national committee for confidential enquiries into maternal deaths. South African Med J. 2015;105(4):287–91.

27. Briand V, Dumont A, Abrahamowicz M, Sow A, Traore M, Rozenberg P, et al. Maternal and Perinatal Outcomes by Mode of Delivery in Senegal and Mali: A Cross-Sectional Epidemiological Survey. PLoS One. 2012;7(10):6–13.

28. Hughes NJ, Namagembe I, Nakimuli A, Sekikubo M, Moffett A, Patient CJ, et al. Decision-to-delivery interval of emergency cesarean section in Uganda: A retrospective cohort study. BMC Pregnancy Childbirth. 2020;20(1):1–10.

29. Goldenberg RL, McClure EM, Bann CM. The relationship of intrapartum and antepartum stillbirth rates to measures of obstetric care in developed and developing countries. Acta Obstet Gynecol Scand.

For peer review only

For peer review only - http://bmjopen.bmj.com/site/about/guidelines.xhtml

2007;86(11):1303–9.

30. van Duinen AJ, Westendorp J, Kamara MM, Forna F, Hagander L, Rijken MJ, et al. Perinatal outcomes of cesarean deliveries in Sierra Leone: A prospective multicenter observational study. Int J Gynecol Obstet. 2020;150(2):213–21.

31. Okonofua FE, Ntoimo LFC, Ogu R, Galadanci H, Mohammed G, Adetoye D, et al. Prevalence and determinants of stillbirth in Nigerian referral hospitals: A multicentre study. BMC Pregnancy Childbirth. 2019;19(1):1–9.

32. Dekker L, Houtzager T, Kilume O, Horogo J, van Roosmalen J, Nyamtema AS. Caesarean section audit to improve quality of care in a rural referral hospital in Tanzania. BMC Pregnancy Childbirth. 2018;18(1):1–7.

33. Sun M, Patauli D, Bernstein PS, Goffman D, Nathan LM. Use of a cesarean delivery checklist in an African maternity ward to improve management and reduce length of hospital stay. Int J Gynecol Obstet. 2020;(January):1–6.

34. Nolens B, Namiiro F, Lule J, van den Akker T, van Roosmalen J, Byamugisha J. Prospective cohort study comparing outcomes between vacuum extraction and second-stage cesarean delivery at a Ugandan tertiary referral hospital. Int J Gynecol Obstet. 2018;142(1):28–36.

35. Litorp H, Kidanto HL, Rööst M, Abeid M, Nyström L, Essén B. Maternal near-miss and death and their association with caesarean section complications: A cross-sectional study at a university hospital and a regional hospital in Tanzania. BMC Pregnancy Childbirth. 2014;14(1):1–10.

36. Chu K, Cortier H, Maldonado F, Mashant T, Ford N, Trelles M. Cesarean Section Rates and Indications in Sub-Saharan Africa: A Multi-Country Study from Medecins sans Frontieres. PLoS One. 2012;7(9):5–10.

37. Bishop D, Dyer RA, Maswime S, Rodseth RN, van Dyk D, Kluyts HL, et al. Maternal and neonatal outcomes after caesarean delivery in the African Surgical Outcomes Study: a 7-day prospective observational cohort study. Lancet Glob Heal. 2019;7(4):e513–22.

38. Madaj B, Smith H, Mathai M, Roos N, Van Den Broek N. Developing global indicators for quality of maternal and newborn care: A feasibility assessment. Bull World Health Organ. 2017;95(6):445-452I.

39. Boatin AA, Schlotheuber A, Betran AP, Moller AB, Barros AJD, Boerma T, et al. Within country inequalities in caesarean section rates: Observational study of 72 low and middle income countries. BMJ. 2018;360.

For peer review only

FIGURES CAPTION LIST

Figure 1: Flow diagram describing the final analytic samples and exclusions.

Figure 2: ***Adjusted odds ratios (aOR and 95% Confidence Interval) for facility-based pregnancy related death and cesarean section stratified by birth outcome and referral status.***[!]
! Adjusted for birth weight, annual delivery volume, type of facility, country and exposure to PTBi intervention. Note – After adjusting for multiple comparison using False Discovery Rate method, the interaction p-values changed to 0.0003 and 0.29, respectively.

Figure 3: Adjusted odds ratios (aOR and 95% Confidence Interval) for adverse neonatal outcomes and cesarean section stratified by referral status.[!]
! Adjusted for birth weight, annual delivery volume, type of facility, country and exposure to PTBi intervention. Note – After adjusting for multiple comparison using False Discovery Rate method, the interaction p-values changed to 0.04 for stillbirth and remained unchanged at 0.85 for predischarge mortality.

For peer review only - http://bmjopen.bmj.com/site/about/guidelines.xhtml

Table 1. Facility, maternal and neonatal characteristics for vaginal deliveries and cesarean sections.

FACILITY CHARACTERISTICS	Vaginal Delivery % (n)	Cesarean Section % (n)	Odds Ratio (95% CI)	p-value (trend)
**Facility type***				
Public	78.7 (50,049)	21.3 (13,572)	1.00	<0.001
Private	62.4 (9,247)	37.7 (5,583)	3.07 (1.71, 5.51)	
**Exposure to PTBi intervention***				
No	75.7 (50,378)	24.4 (16,218)	1.00	0.455
Yes	75.3 (8,918)	24.8 (2,937)	1.51 (0.51, 4.46)	
**Annual delivery volume***				
< 1000 (Low)	68.4 (918)	31.7 (425)	1.46 (0.65, 3.28)	0.594
1000 – < 2800 (Medium)	73.0 (16,890)	27.0 (6,251)	1.00	
≥ 2800 (High)	76.9 (41,488)	23.1 (12,479)	0.76 (0.28, 2.04)	

MATERNAL & NEONATAL CHARACTERISTICS	Vaginal Delivery % (n)	Cesarean Section % (n)	Odds Ratio (95% CI)	p-value
**Maternal age (years)***				
< 18	76.4 (3,788)	23.6 (1,168)	1.06 (0.90, 1.26)	0.823
18 – ≤ 35	75.7 (51,924)	24.3 (16,686)	1.00	
36 – 53	74.0 (3,151)	26.1 (1,110)	1.04 (0.93, 1.16)	
**Delivery referred in***				
No	75.5 (32,823)	24.5 (10,661)	1.00	0.17
Yes	55.0 (3,847)	45.0 (3,146)	2.33 (2.06, 2.64)	
Missing	80.9 (22,542)	19.1 (5,324)	0.98 (0.83, 1.15)	
**Day of delivery***				
Monday – Friday	75.2 (41,781)	24.7 (13,773)	1.00	0.009
Weekend	76.7 (16,735)	23.3 (5,086)	0.93 (0.88, 0.98)	
**Maternal length of stay ***				
< 7 days	76.7 (57,593)	23.3 (17,462)	1.00	<0.001
≥ 7 days	33.4 (543)	66.6 (1,083)	1.93 (1.82, 2.04)	
**Multiple gestation***				
Singleton	75.9 (57,717)	24.1 (18,286)	1.00	<0.001
Twins, triplets or quadruplets	64.5 (1,579)	35.5 (869)	1.77 (1.53, 2.04)	
**Preterm birth (<37 completes weeks)***				
No	75.9 (52,493)	24.1 (16,708)	1.00	0.016
Yes	73.6 (6,803)	26.5 (2,447)	1.08 (1.01, 1.15)	
**Gestational age (weeks)***				
20 – < 28	85.8 (338)	14.2 (56)	0.57 (0.41, 0.80)	<0.001
28 – < 32	80.8 (1,337)	19.2 (318)	0.74 (0.60, 0.91)	
32 – < 37	74.1 (5,170)	25.9 (1,809)	1.14 (1.03, 1.25)	
37 – < 40	75.7 (34,303)	24.3 (10,986)	1.00	
≥ 40	76.2 (8,811)	23.8 (2,754)	1.08 (1.01, 1.15)	
**Low birth weight (< 2500 grams)****				
No	75.9 (52,819)	24.1 (16,777)	1.00	<0.001
Yes	71.1 (5,926)	28.9 (2,405)	1.22 (1.12, 1.33)	
**Birth weight (grams)****				
< 1000	89.9 (257)	10.1 (29)	0.34 (0.18, 0.66)	<0.001
1000 – 2499	71.1 (5,926)	28.9 (2,405)	1.24 (1.14, 1.35)	
2500 – 3999	76.2 (50,006)	23.8 (15,599)	1.00	
≥ 4000	70.5 (2,813)	29.5 (1,178)	1.39 (1.25, 1.55)	
**Apgar score at 5 minutes ****				
≥ 7	75.7 (47,154)	24.3 (15,159)	1.00	<0.001
< 7	63.0 (2,135)	37.0 (1,255)	1.78 (1.31,2.40)	

** Based on the delivery dataset of 78,451 mothers; ** Based on the neonatal dataset of 80,976*

For peer review only

Table 2. Adjusted[!] odds ratios for neonatal and maternal outcomes amongst those who had cesarean sections.

	Vaginal Delivery % (n)	Cesarean Section % (n)	Adjusted Odds Ratio (95% CI)	P-value	Adjusted P-value[@]
**Stillbirth ***					
No	75.5 (58,338)	24.5 (18,904)	1.00	0.006	0.01
Yes	69.0 (2,576)	31.0 (1,158)	1.33 (1.09, 1.63)		
**Pre-discharge neonatal mortality ***					
No	75.4 (60,174)	24.6 (19,606)	1.00	<0.001	0.0003
Yes	61.9 (740)	38.1 (456)	1.88 (1.64, 2.16)		
**Maternal mortality ****					
No	75.2 (52,694)	24.8 (17,419)	1.00	<0.001	0.0003
Yes	49.4 (77)	50.6 (79)	3.28 (2.21, 4.88)		

! Adjusted for birth weight, annual delivery volume, type of facility and country and exposure to PTBi intervention.
[@]P-values adjusted for multiple comparison using False Discovery Rate method.
** Based on the neonatal dataset of 80,976*
*** Based on the delivery dataset of 78,451 mothers*

For peer review only

For peer review only - http://bmjopen.bmj.com/site/about/guidelines.xhtml

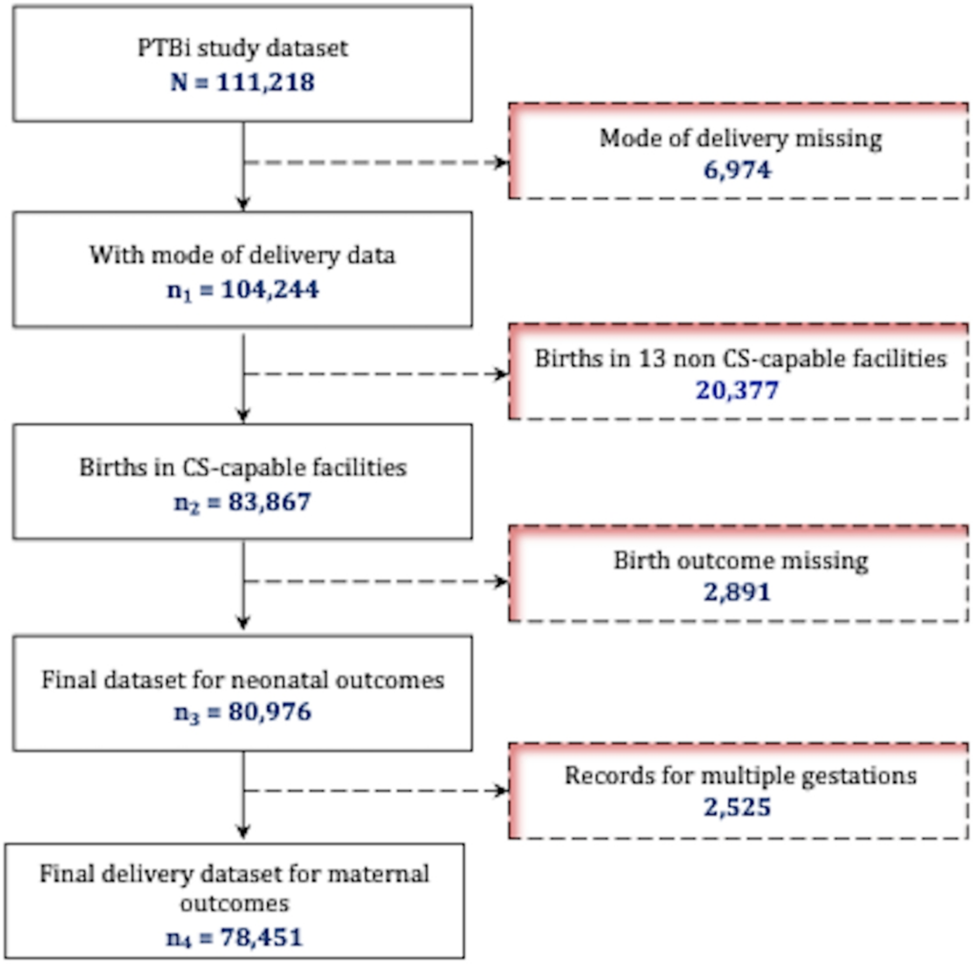

Figure 1: Flow diagram describing the final analytic samples and exclusions.

133x133mm (300 x 300 DPI)

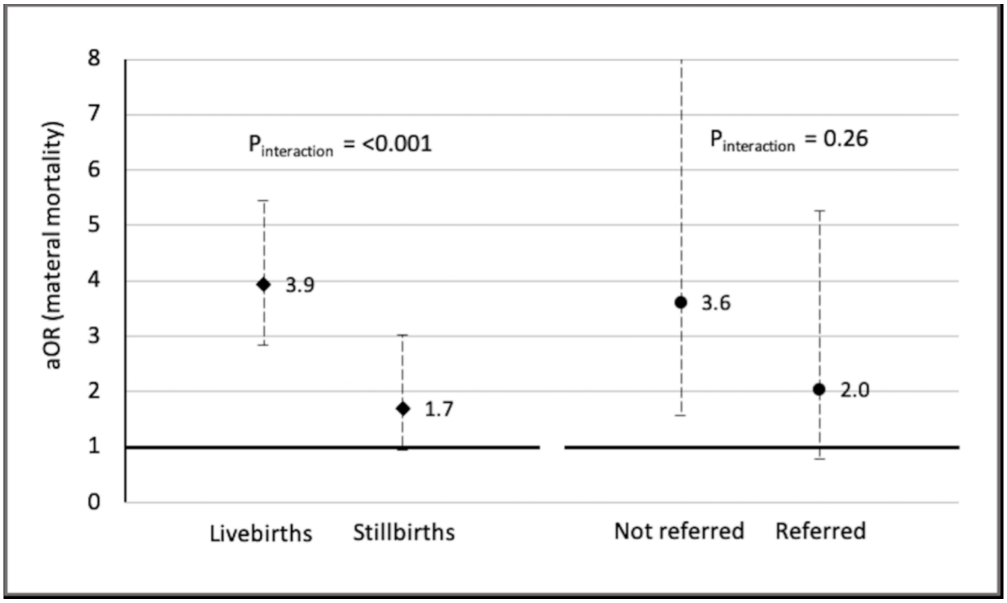

Figure 2: Adjusted odds ratios (aOR and 95% Confidence Interval) for facility-based pregnancy related death and cesarean section stratified by birth outcome and referral status.!

137x82mm (300 x 300 DPI)

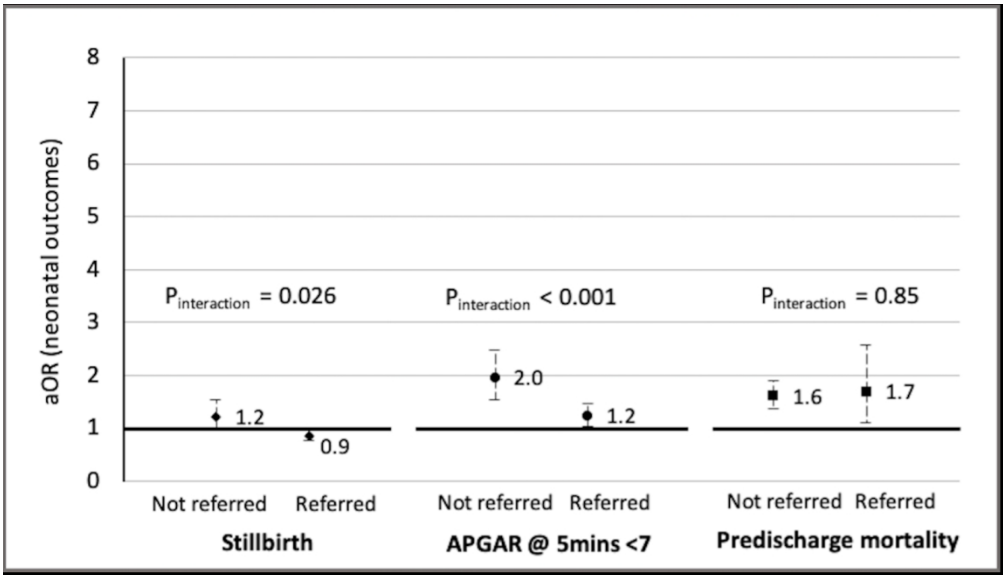

Figure 3: Adjusted odds ratios (aOR and 95% Confidence Interval) for adverse neonatal outcomes and cesarean section stratified by referral status.!

137x78mm (300 x 300 DPI)

Online supplement

The hypothesized relationship is between cesarean section and maternal mortality as shown by the thick red arrow in the online supplementary figure S1. To keep the conceptual framework simple, we are showing the Directed Acyclic Graph (DAG) for one outcome only. We considered each potential confounder and discussed all the backdoor pathways connecting the exposure (cesarean section) to the outcome (maternal mortality) through the confounders. In other words, once that confounder is adjusted, those pathways will no longer exist, potentially eliminating confounding, estimating only the *direct effect* between exposure and the outcome. We assume that there is no confounder misclassification i.e., after adjustment for the confounder in the model there is no residual confounding left from that confounder. Further, the majority of these confounders are categorical, and in accordance with their biological relationship with the exposure and the outcome. Hence, their functional form is assumed to be adequate to account for confounding.

1. Adjusting for *multiple gestation* would eliminate all the backdoor paths connecting mortality to cesarean section, through PTBi intervention. The backdoor paths from viability and birth weight/gestational age (GA) through multiple gestation are also removed. In the backdoor path MMR – multiple gestation – cesarean section – birth weight/GA, cesarean section is a collider. Adjustment for multiple gestation is necessary because multiple gestation pregnancy is a clinical indication for cesarean section. However, this could lead to over adjustment by inflating the standard error, as cesarean section is a collider in this pathway. We will still include multiple gestation in the preliminary model.
2. Adjusting for *birth weight/GA* would eliminate the direct path from birth weight/ GA to cesarean section and the backdoor paths through PTBi intervention, maternal history (e.g., age, prior cesarean section), viability, complications that leads to maternal deaths. The pathway from birth weight/GA – complications – maternal history leading on to maternal deaths through several other variables in the DAG are blocked paths because complication is a collider. Irrespective, birth weight adjustment removes the direct path from cesarean section – birth weight/GA – complications and will be included in the model.
3. Adjusting for *delivery volume*, will remove the backdoor from MMR – delivery volume – cesarean section. Further, it removes two additional directed paths from delivery volume to facility type and country and will be included in the model.
4. *PTBi intervention* requires adjustment because of the backdoor path from maternal death – PTBi – cesarean section. It will also remove the directed paths from PTBi intervention – facility readiness, PTBi intervention – APGAR score, facility type – PTBi intervention and country – PTBi intervention.
5. *Facility type* requires adjustment for the backdoor path maternal death – facility type – cesarean section. This adjustment will also remove the directed path from country – facility type.
6. The last confounder necessary to adjust is *country* for the backdoor path MMR – country – cesarean section.
7. *Maternal complications* will determine the need for a cesarean section. Independent of those cases that require a cesarean section, maternal complications such as PPH are primary reasons for maternal deaths. Complete maternal complication data is unavailable and hence it is a limitation. Statistically it leaves the potential for residual confounding.
8. A close look at this stage of the model will reveal that *APGAR score* does not require adjustment. All the backdoor paths from maternal death to cesarean section are through APGAR and stillbirth, making APGAR a collider and all those are blocked paths.
9. All other paths from viability, *weekend delivery and facility readiness* are blocked paths where cesarean section is the collider. Hence, conceptually there is no need for adjustment of any other confounder.
10. Of birth weight and GA, the final model had birth weight and not both because including GA leads to about loss of 8000 births, both are colinear (Pearson's correlation = 0.5) and the AICs for both models are close (AIC for the birth weight model is 1779 and AIC for the GA model is 1693).
11. Statistically adjusting for multiple gestation did not change the exposure – outcome association. The association with MMR presented in table 2 changed to 3.31 (2.21, 4.97) after including multiple gestation in the reported model. Hence, multiple gestation was not retained in the final model.

For peer review only - http://bmjopen.bmj.com/site/about/guidelines.xhtml

Figure S1. Conceptual diagram describing the hypothesized relationship (red arrow) between cesarean section and maternal mortality and the potential confounding due to other factors.

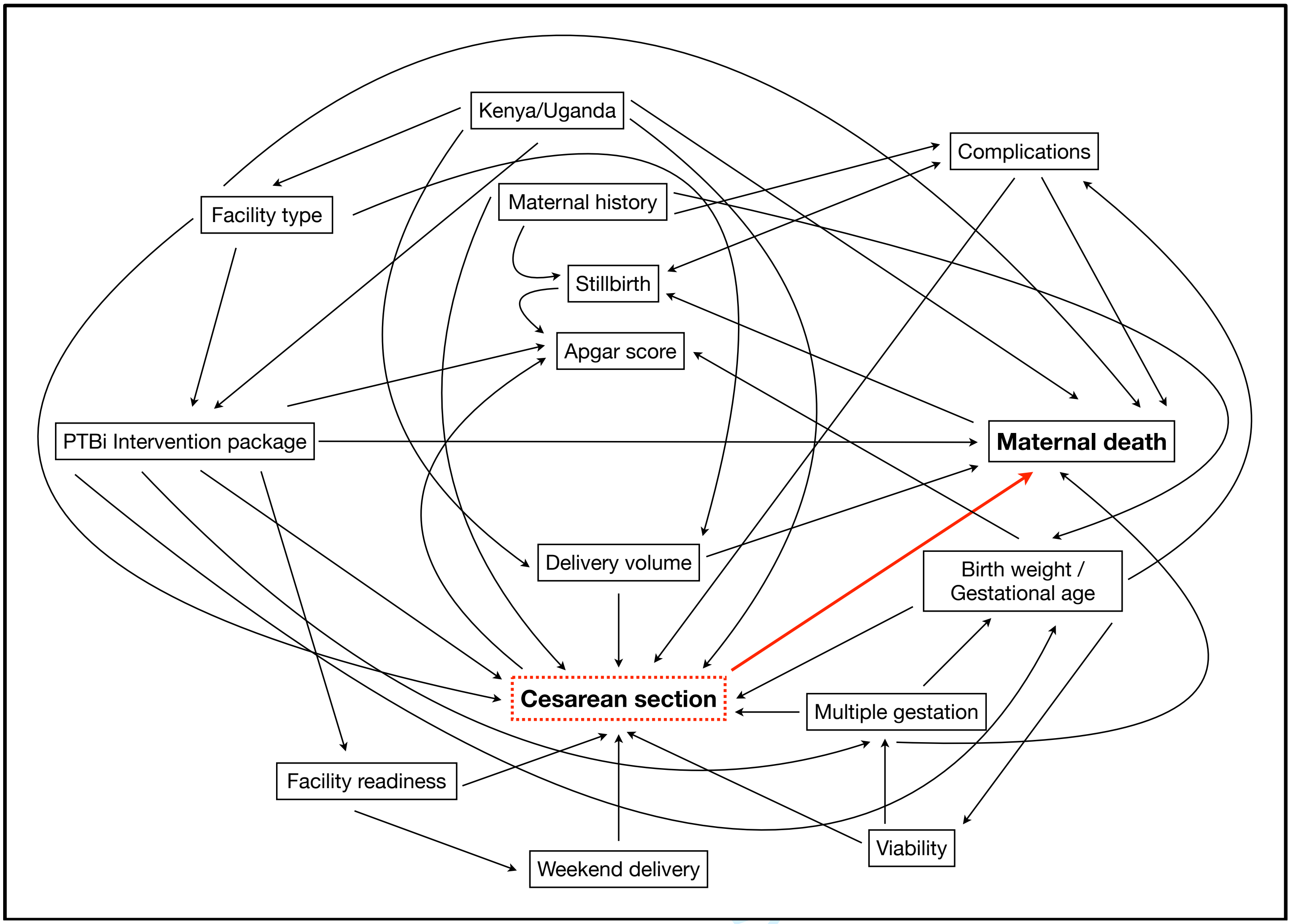

For peer review only - http://bmjopen.bmj.com/site/about/guidelines.xhtml

Table S1. Adjusted[!] odds ratios for types of stillbirths and cesarean sections.

	Vaginal Delivery % (n)	Cesarean Section % (n)	Adjusted Odds Ratio (95% CI)	P-value
**Antepartum/macerated stillbirth ***				
Live birth	75.7 (56,718)	24.3 (18,193)	1.00	
Yes	79.6 (1,200)	20.4 (308)	0.74 (0.65, 0.84)	<0.001
**Intrapartum/fresh stillbirth ***				
Live birth	75.7 (56,718)	24.3 (18,193)	1.00	
Yes	60.4 (1,084)	39.6 (710)	1.79 (1.63, 1.98)	<0.001

! Adjusted for birth weight, annual delivery volume, type of facility, country and exposure to PTBi intervention. The models are single-level logistic regression.
** Based on the neonatal dataset of 80,976*

For peer review only

Table S2. Associations between pregnancy related maternal death and cesarean section stratified by birth outcome and referral status.[!]

		**Maternal mortality** (C Section × Stillbirths, $P_{interaction}$ <0.001*)		**Maternal mortality** (C Section × Referral Status, $P_{interactions}$ = 0.26 and 0.80*)		
		**Livebirths**	**All stillbirths**	**Not referred**	**Referred**	**Missing**
	n	66,098	2,954	39,519	6,482	21,472
**C Section** **	No	1.00	1.00	1.00	1.00	1.00
	Yes	3.94 (2.84, 5.46)	1.70 (0.95, 3.03)	3.61 (1.56, 8.37)	2.04 (0.79, 5.27)	3.10 (2.06, 4.65)
	FDR Unadjusted p-value	<0.001	0.07	0.003	0.14	0.001
	FDR adj p-value	0.0003	0.09	0.006	0.16	0.002
**Number of events**						
**C Section**	No	32	37	28	8	31
	Yes	44	25	33	12	22

! Adjusted for birth weight, annual delivery volume, type of facility and country.

** Interaction between the two variables and the p-value for the interaction term. For Referral Status, two p-values are reported because the variable has 3 categories. After adjusting for multiple comparison using False Discovery Rate (FDR) method, the FDR unadjusted interaction p-values changed from <0.001 to 0.0003 for stillbirth and from 0.26 to 0.29 and 0.80 to 0.83 for referral status..*

For peer review only

For peer review only - http://bmjopen.bmj.com/site/about/guidelines.xhtml

Table S3. Associations between adverse neonatal outcomes and cesarean section stratified by referral status.[!]

	**Stillbirth** (C Section × Referral Status, $P_{interaction}$ = 0.026 and 0.046*)			**Apgar <7** (C Section × Referral Status, $P_{interaction}$ <0.001 and 0.69*)			**Pre-discharge neonatal mortality** (C Section × Referral Status, $P_{interaction}$ = 0.85 and 0.003*)		
	**Not referred**	**Referred**	**Missing**	**Not referred**	**Referred**	**Missing**	**Not referred**	**Referred**	**Missing**
n	42,813	7,007	27,625	35,075	6,218	22,651	43,349	7,110	27,625
**C Section**** No	1.00	1.00	1.00	1.00	1.00	1.00	1.00	1.00	1.00
Yes	1.22 (0.96, 1.54)	0.86 (0.77, 0.97)	1.74 (1.22, 2.46)	1.96 (1.55, 2.48)	1.24 (1.05, 1.47)	2.02 (1.41, 2.89)	1.61 (1.37, 1.90)	1.69 (1.11, 2.59)	2.31 (1.82, 2.93)
FDR Unadj pval	0.10	0.012	0.002	<0.001	0.01	<0.001	<0.001	0.014	<0.001
FDR adj p-value	0.12	0.019	0.005	0.0003	0.02	0.0003	0.003	0.021	0.003
No. of events									
C Section. No	1,068	337	879	682	293	974	353	87	234
Yes	445	241	332	471	303	413	193	108	124

! Adjusted for birth weight, annual delivery volume, type of facility and country.

** Interaction between the two variables and the p-values for the interaction terms. Two p-values are reported because the referral variable has 3 categories. After adjusting for multiple comparison using False Discovery Rate (FDR) method, the interaction p-values changed to 0.04 and 0.06 for stillbirth, 0.0003 and 0.74 for Apgar and 0.85 and 0.006 for predischarge mortality. These p-values should be compared to the ones in the very top row.*

For peer review only

STROBE Statement—Checklist of items that should be included in reports of ***cohort studies***

	Item No	Recommendation	Page number(s)
**Title and abstract**	1	(*a*) Indicate the study's design with a commonly used term in the title or the abstract	1
		(*b*) Provide in the abstract an informative and balanced summary of what was done and what was found	2
**Introduction**			
Background/rationale	2	Explain the scientific background and rationale for the investigation being reported	4
Objectives	3	State specific objectives, including any prespecified hypotheses	4 and 5
**Methods**			
Study design	4	Present key elements of study design early in the paper	5
Setting	5	Describe the setting, locations, and relevant dates, including periods of recruitment, exposure, follow-up, and data collection	5
Participants	6	(*a*) Give the eligibility criteria, and the sources and methods of selection of participants. Describe methods of follow-up	6 and Figure 1
		(*b*) For matched studies, give matching criteria and number of exposed and unexposed	Not applicable
Variables	7	Clearly define all outcomes, exposures, predictors, potential confounders, and effect modifiers. Give diagnostic criteria, if applicable	6
Data sources/ measurement	8*	For each variable of interest, give sources of data and details of methods of assessment (measurement). Describe comparability of assessment methods if there is more than one group	6
Bias	9	Describe any efforts to address potential sources of bias	14 (limitation section)
Study size	10	Explain how the study size was arrived at	8 and Figure 1
Quantitative variables	11	Explain how quantitative variables were handled in the analyses. If applicable, describe which groupings were chosen and why	6, 7 and the DAG shown in supplementary figure S1
Statistical methods	12	(*a*) Describe all statistical methods, including those used to control for confounding	7 and DAG, Figure S1
		(*b*) Describe any methods used to examine	7

For peer review only - http://bmjopen.bmj.com/site/about/guidelines.xhtml

For peer review only

		subgroups and interactions	
		(*c*) Explain how missing data were addressed	8
		(*d*) If applicable, explain how loss to follow-up was addressed	Not applicable
		(*e*) Describe any sensitivity analyses	Not applicable
**Results**			
Participants	13*	(a) Report numbers of individuals at each stage of study—eg numbers potentially eligible, examined for eligibility, confirmed eligible, included in the study, completing follow-up, and analysed	8,9 and Figure 1
		(b) Give reasons for non-participation at each stage	Figure 1
		(c) Consider use of a flow diagram	Figure 1
Descriptive data	14*	(a) Give characteristics of study participants (eg demographic, clinical, social) and information on exposures and potential confounders	Table 1
		(b) Indicate number of participants with missing data for each variable of interest	Figure 1 and Table 1
		(c) Summarise follow-up time (eg, average and total amount)	Not applicable
Outcome data	15*	Report numbers of outcome events or summary measures over time	9
Main results	16	(*a*) Give unadjusted estimates and, if applicable, confounder-adjusted estimates and their precision (eg, 95% confidence interval). Make clear which confounders were adjusted for and why they were included	9 and 10. Table 2. Footnotes in table 2 state the variables adjusted
		(*b*) Report category boundaries when continuous variables were categorized	Table 1
		(*c*) If relevant, consider translating estimates of relative risk into absolute risk for a meaningful time period	Not applicable
Other analyses	17	Report other analyses done—eg analyses of subgroups and interactions, and sensitivity analyses	10, Figure 2
**Discussion**			
Key results	18	Summarise key results with reference to study objectives	10 and 11
Limitations	19	Discuss limitations of the study, taking into account sources of potential bias or imprecision. Discuss both direction and magnitude of any potential bias	14
Interpretation	20	Give a cautious overall interpretation of results considering objectives, limitations,	11 and 14

For peer review only - http://bmjopen.bmj.com/site/about/guidelines.xhtml

		multiplicity of analyses, results from similar studies, and other relevant evidence	
Generalisability	21	Discuss the generalisability (external validity) of the study results	14
**Other information**			
Funding	22	Give the source of funding and the role of the funders for the present study and, if applicable, for the original study on which the present article is based	16

*Give information separately for exposed and unexposed groups.

For peer review only
